# Teeth morphology in Red Sea fishes and comparative evolution of tooth-associated genes in herbivorous and carnivorous

**DOI:** 10.1186/s12983-026-00635-0

**Published:** 2026-11-02

**Authors:** Eman E. El-Nahass, Mohamed Nagy, Aya E. Khalifa

**Affiliations:** Zoology Department, Faculty of Science, Tanta University, Tanta, 31527 Egypt

**Keywords:** Red Sea fish, Phylogenetic trees, Adaptive evolution, Herbivory and carnivory fish, Odontogenesis, Positive selection

## Abstract

Fish dentition exhibits substantial variability that reflects ecological niches and dietary specialization within the Red Sea ecosystem, highlighting adaptive evolutionary responses to different feeding strategies. This study investigated tooth morphology using scanning electron microscopy and explored the evolutionary basis of dental diversity through comparative genomic analyses. Ultrastructural examination of six representative fish families revealed distinct dentition patterns. Herbivorous (Scaridae and Siganidae) displayed incisiform, villiform, and molariform teeth, whereas carnivorous (Sphyraenidae, Holocentridae, Serranidae, Lethrinidae, and Lutjanidae) exhibited sharp conical and canine-like dentition adapted for prey capture. In parallel, 130 tooth-associated genes were analyzed across 25 teleost genomes representing herbivorous and carnivorous lineages. Phylogenetic analyses recovered well-supported family-level clades and suggested independent origins of herbivory consistent with convergent evolution. Selection analyses detected widespread episodic positive selection, with 70 genes remaining significant after correction. Branch-level analyses further identified reciprocal selection patterns between herbivorous and carnivorous lineages. Site-level analyses detected adaptive codons overlapping predicted functional regions in lrp5 and pam. Comparative tests further revealed contrasting selection pressures between trophic groups, with genes related to odontogenesis and skeletal development intensified in herbivores, whereas genes associated with growth factor signaling and neural processes were intensified in carnivores. Together, these findings highlight the interplay between dietary ecology, tooth morphology, and molecular evolution in teleost fishes. Functional validation will be required to confirm these genomic predictions.

**Supplementary information:**

The online version contains supplementary material available at https://doi.org/10.1186/s12983-026-00635-0.

## Introduction

The Red Sea is one of the most biodiverse marine environments of the world, hosting a vast number of fish species that exhibit unique ecological and morphological adaptations. Feeding structures, particularly teeth, can reveal information about an organism’s ecology, including the distribution of its habitat, as well as its feeding habits. Dental morphology is often closely related with the types of food eaten, and variations in tooth structure reflect adaptations to specific feeding strategies [1]. Teeth represent the hardest tissue in most living vertebrates and play a key role in prey capture and masticate food. Unlike humans, which have two sets of teeth (diphyodont, changing teeth only once), fish are thought to be polyphyodont (constantly replacing their teeth) [2–4]. Fish species have evolved variety of tooth forms, shapes, and arrangements depending on dietary habits and ecological niche [5, 6]. This diversity includes small or large teeth that may be conical, villiform, or molariform [7]. Furthermore, the distribution of oral teeth throughout the jaw varies greatly among fishes compared with mammals [7, 8].

Based on diet, fish can be classified into several major trophic groups: herbivores, omnivores, detritivores, mesocarnivores, and apex predators, alongside highly specialized feeders like crustacivores and molluscivores [8]. These feeding strategies are reflected in differences in tooth structure and function [9, 10]. For example, herbivorous species such as the surgeonfish (*Acanthurus sohal*) possess teeth adapted for scraping algae [11, 12], whereas fishes that feed on hard-shelled prey such as mollusks and crustaceans often possess strong, compact teeth capable of crushing resistant food items. The benthic invertebrate-eating rock mover wrasse (*Novaculichthys taeniourus*) overturns rocks with its strong jaws in order to get at its prey [13]. Coral fish have fine mouths with fine teeth and a sharp cutter bite for coral. The teeth of carnivorous fish are made to puncture, hold, and cut their prey [8, 13]. The great barracuda *(Sphyraena barracuda*), an apex predator found in tropical seas around the world, eats almost exclusively other fish [12, 14]. Its large gape and extremely sharp teeth allow it to chop large fish in half, earning it the nickname “Tiger of the Sea” [15]. In several teleost lineages, such as Cichliformes and Labridae, pharyngeal teeth-situated within the pharynx immediately anterior to the esophagus-work in functional cooperation with oral jaw teeth to facilitate prey processing and swallowing [8, 16]. These pharyngeal teeth and jaws work together to process food by crushing and masticating before transporting it to the esophagus for swallowing [16, 17]. 

Although dental morphology is a cornerstone of trophic ecology, high-resolution quantitative insights into the ultrastructural characteristics of marine teleost dentition remain remarkably scarce. To address this fundamental gap and elucidate the fine-scale ecomorphological adaptations driving dietary specialization, this study utilizes scanning electron microscopy (SEM) to systematically analyze the dental ultrastructure of several representative species. The aims were to characterize tooth morphology and classify dentition types in relation to feeding strategies. In addition, a comparative genomic analysis of tooth-associated genes was conducted across representative taxa to explore evolutionary patterns underlying dental diversity. By integrating morphological observations with genomic data, this study aims to provide insights into how dietary specialization and ecological pressures shape the evolution and diversity of dentition in Red Sea fishes.

## Material and methods

### Fish sampling collection

**Fig. 1:**
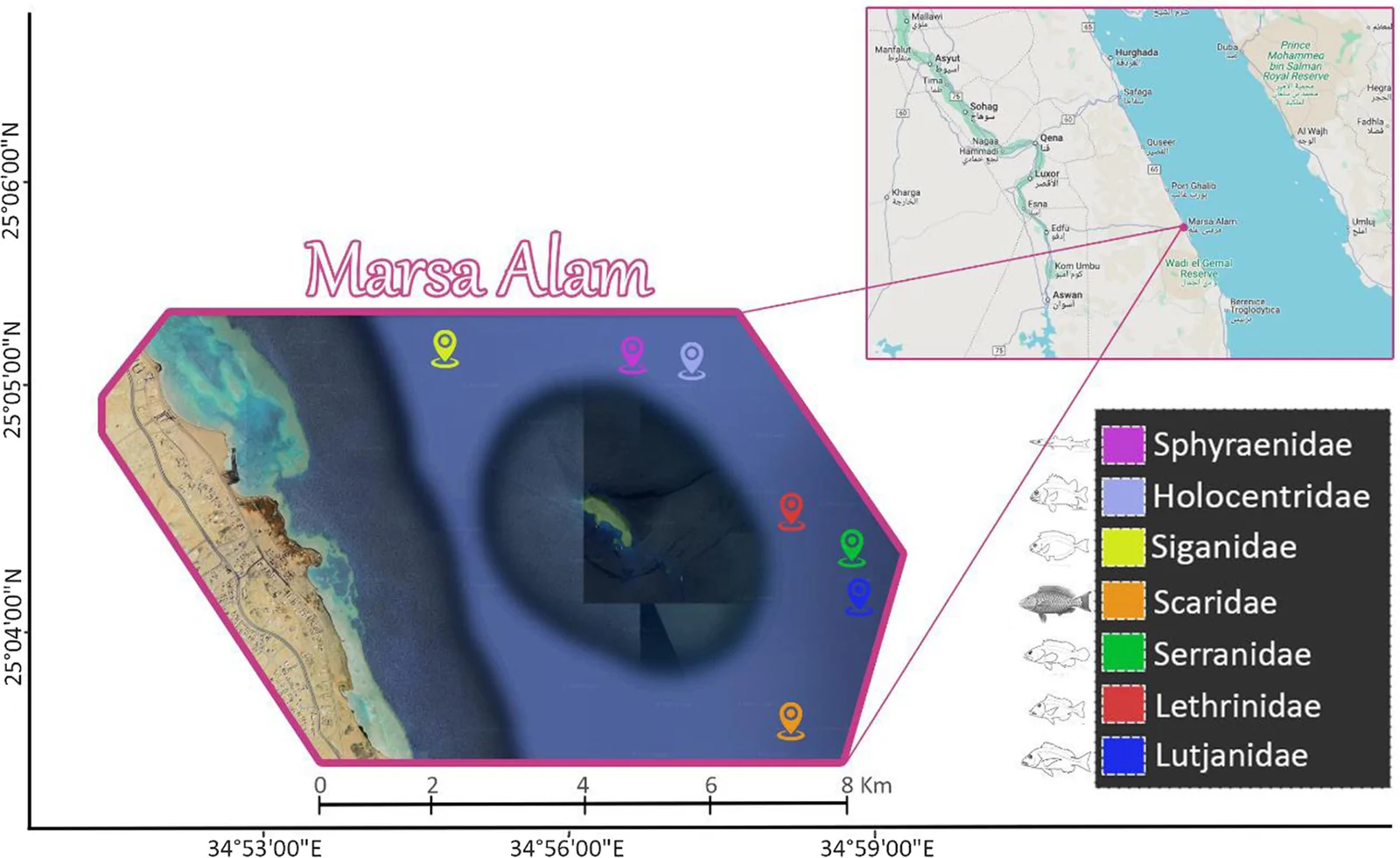
A geographic map showing the sampling localities and spatial distribution of selected fish families in the *Marsa Alam* marine region, Red Sea

Twelve adult specimens from each studied species were used in this study (*Chlorurus sordidus*, *Scarus ghobban*, *Scarus psittacus*, *Siganus fuscescens*, *Siganus stellatus*, *Siganus canaliculatus*, *Sphyraena obtusata*, *Sargocentron spiniferum*, *Cephalopholis miniata*, *Lethrinus lentjan*, *Lethrinus nebulosus*, and *Lutjanus rivulatus*) (Fig. 1). These species were collected from Egyptian Red Sea in August. The specimens were brought to our lab within eight hours in a plastic aquarium for gross examination and SEM analysis of the tooth structure. All fishes were anesthetized using benzocaine (4 mg/L) prior to examination. Sample collection followed the “Sampling protocol for the pilot collection of catch, effort, and biological data in Egypt” [18].

### Gross anatomy

For the gross anatomical analysis, three specimens from each species were examined. After dissected, the upper and lower jaws as well as the oropharyngeal cavities were carefully removed, cleaned with regular saline, and inspected for any damage or anomalies. The general anatomical characteristics were described, and photographic documentation of the jaws and pharyngeal structures was obtained using a stereoscopic microscope. The dissected jaws were then fixed in 10% neutral buffered formalin (NBF) in labeled containers for further analysis.

### Stereoscopic examination

The upper and lower jaws as well as the oropharyngeal cavities of all studied fish species were examined using a Stemi 508 stereomicroscope (Carl Zeiss, Germany). The photomicrographs were captured using a Canon Powershot A95 camera connected to a Microscopy GmbH at the Faculty of Medicine, Tanta University, Egypt.

### Morphometric measurements

**Fig. 2:**
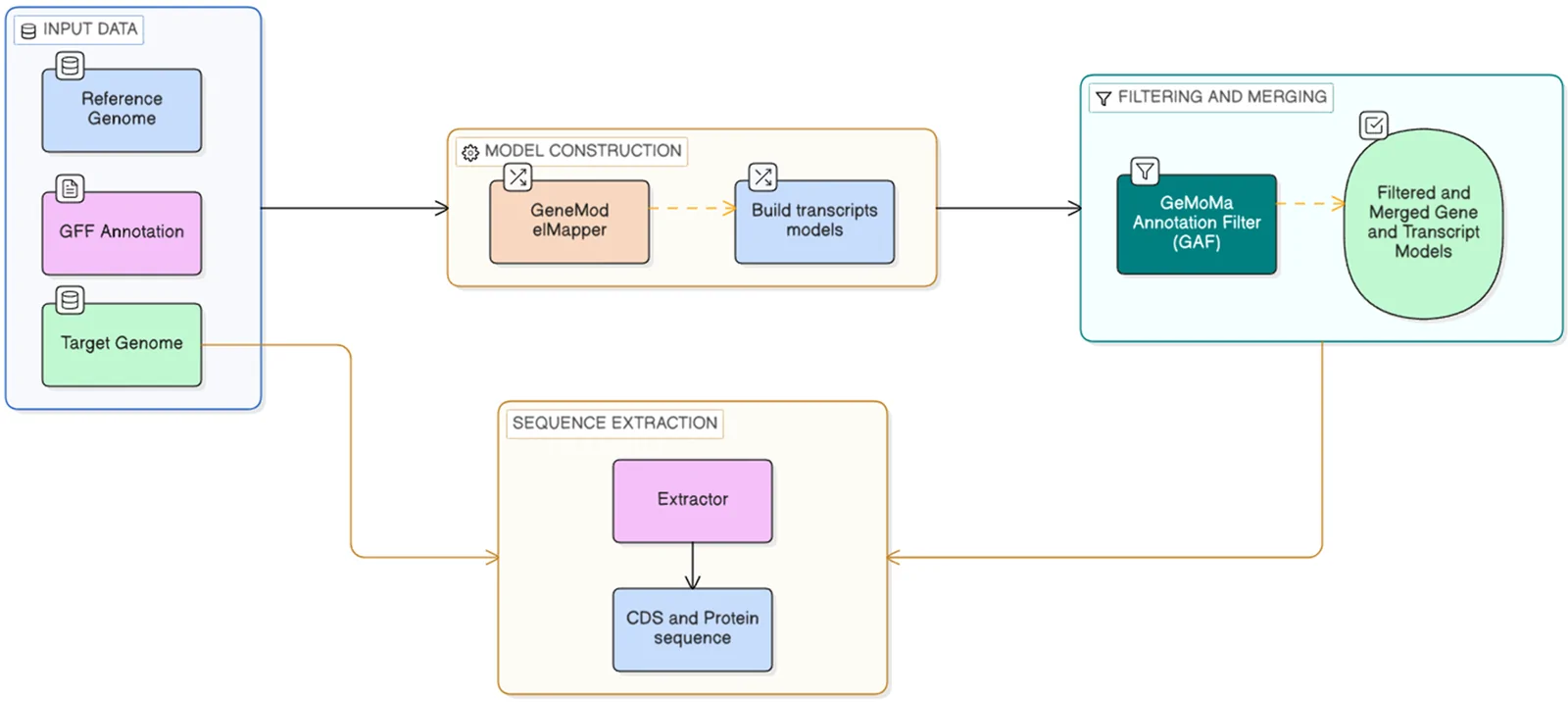
Workflow diagram of homology-based gene prediction using gene model mapper GeMoMa, merging predictions with GeMoMa annotation filter GAF, and extraction of CDS and protein sequences with GeMoMa extractor

Two digital scales were used for the gross morphological measurements, which included fish body weight (g) and jaw weight (g) (Fig. 2). The sensitivity of the first scale was 1–1 kg, while the sensitivity of the second scale was 0.01–1 g. A digital Vernier caliper was also used to measure the length and width of the fish body, fins, jaws, and pharynx, all measurements were recorded in Table 1.

**Table 1:** The gross morphological measurements of studied fish species in the *Marsa Alam* marine region, Red Sea

No	Species	Average body weight ±SD	Average jaws weight ±SD (gm)		Average pharynx weight ±SD	Average body length ±SD	Average jaws length±SD (cm)		Average pharynx length ±SD
			Upper jaw	Lower jaw			Upper jaw	Lower jaw	
1	*C. sordidus*	109.66 ± 5.5^f^	9.27 ± 0.34^c^	2.19 ± 0.07^e^	2.21 ± 0.09	20.66 ± 1.52^b^	4.04 ± 0.07^e^	3.87 ± 0.12^d^	1.93 ± 0.07^e^
2	*S. ghobban*	158.33 ± 7.76^d^	5.07 ± 0.13	2.50 ± 0.03^e^	2.5 ± 0.14^e^	21.66 ± 1.53^b^	4.99 ± 0.12^c^	4.15 ± 0.15^d^	2.54 ± 0.10^d^
3	*S. psittacus*	149.66 ± 2.08^e^	9.92 ± 0.17^c^	1.54 ± 0.18	1.52 ± 0.13	21.33 ± 1.53^b^	4.92 ± 0.08^c^	4.48 ± 0.14^c^	2.55 ± 0.06^d^
4	*S. fuscescens*	149.33 ± 6.5^e^	7.44 ± 0.21^e^	4.63 ± 0.20^c^	2.52 ± 0.11^e^	20.33 ± 1.52^b^	3.83 ± 0.15^e^	2.17 ± 0.08^f^	3.02 ± 0.05^c^
5	*S. stellatus*	109.33 ± 10.4^f^	8.01 ± 0.10^d^	5.05 ± 0.11^c^	3.76 ± 0.07^d^	20.66 ± 1.52^b^	3.49 ± 0.14^f^	2.86 ± 0.10^e^	2.51 ± 0.12^d^
6	*S. canaliculatus*	100.33 ± 4.04^f^	8.56 ± 0.33^d^	2.03 ± 0.07	26.2 ± 0.21^a^	23 ± 1.73^b^	4.42 ± 0.03^d^	3.00 ± 0.02^e^	2.97 ± 0.03^c^
7	*S. obtusata*	699.66 ± 10.01^a^	40.44 ± 0.33^a^	20.18 ± 0.23^a^	10.22 ± 0.100^b^	53 ± 1.73^a^	6.0 ± 0.02^a^	6.91 ± 0.12^a^	4.96 ± 0.11^a^
8	*S. spiniferum*	201.66 ± 11.67^c^	7.17 ± 0.18^f^	4.09 ± 0.20^d^	25.24 ± 0.27^a^	22 ± 1.01^b^	5.03 ± 0.06^c^	4.04 ± 0.07^d^	1.54 ± 0.09^f^
9	*C. miniata*	171.33 ± 3.05^d^	12.10 ± 0.27^b^	7.49 ± 0.28^b^	7.51 ± 0.05^c^	23.33 ± 0.57^b^	5.96 ± 0.14^b^	5.51 ± 0.14^b^	4.01 ± 0.07^b^
10	*L. lentjan*	141.33 ± 4.93^e^	2.68 ± 0.26	2.57 ± 0.29^e^	4.56 ± 0.16^d^	20.66 ± 1.52^b^	5.05 ± 0.14^c^	4.5 ± 0.11^c^	4.02 ± 0.08^b^
11	*L.nebulosus*	113.66 ± 4.5^f^	1.89 ± 0.06	1.87 ± 0.08	2.96 ± 0.06	21.33 ± 1.53^b^	4.08 ± 0.20^d^	4.11 ± 0.10^d^	4.02 ± 0.06^b^
12	*L. rivulatus*	259.33 ± 8.08^b^	3.92 ± 0.11	3.58 ± 0.07^d^	8.04 ± 0.06^c^	23.33 ± 1.52^b^	4.84 ± 0.15^c^	4.58 ± 0.02^c^	5.06 ± 0.12^a^
F-Value		1577.83	5684.26	2486.58	884.24	114.85	163.2	326.93	497.5
*p*-Value		<0.001	<0.001	<0.001	<0.001	<0.001	<0.001	<0.001	<0.001

a, b, c, d, e and f refer to the significance of each letter compared to the following letters. Means that do not share a letter are significantly different (Tukey’s test, *p* < 0.05).

### Scanning electron microscopic investigations

For SEM analysis, three specimens from each fish species were examined. After dissection, the pharynx, upper jaws, and lower jaws were carefully separated and fixed for four hours at 4 °C in a solution of 2.5% paraformaldehyde and 2.5% glutaraldehyde in 0.1 M phosphate buffer. Then post-fixed for two hours in phosphate buffer containing 1% osmium tetroxide. After that, the samples were dehydrated in progressively higher ethanol concentrations before the critical point dried in carbon dioxide [19], then were sputter-coated with gold and examined by a JEOL JSM-IT200 scanning electron microscope at the Faculty of Agriculture, El Mansoura University, Egypt.

### Species selection and genome dataset

To construct a comparative molecular framework for investigating the evolutionary dynamics of tooth-associated genes, the initial dataset of 12 focal species was expanded to a broader phylogenomic panel of 30 teleost fishes. The spotted gar (*Lepisosteus oculatus*) was selected as the out-group due to its phylogenetic position outside Teleostei, provided an appropriate reference for rooting evolutionary analyses. Species selection was based on three main criteria: (i) the availability of high-quality, publicly accessible genome assembly, (ii) taxonomic proximity to the focal taxa, to ensure family-level representation, and (iii) balanced representation of trophic strategies to enable comparative analyses between carnivorous and herbivorous lineages. The final dataset comprised species from eight teleost families: Sphyraenidae, Serranidae, Lethrinidae, Lutjanidae, Siganidae, Acanthuridae, Labridae, and Holocentridae. Trophic classifications (carnivorous or herbivorous) were assigned based on ecological information obtained from Fish Base [20]. In total, the dataset included 13 carnivorous and 17 herbivorous species, in addition to the out-group. Genome assemblies were retrieved from the NCBI Genome database, and the corresponding accession numbers are provided in Table 2. For each species, assembly accession and genome size were recorded.

**Table 2:** Taxonomic identity, dietary classification, genome assembly accession, and genome size for all species included in the study

Family	Organism name	Diet	Genome assembly	Genome size
Sphyraenidae	*Sphyraena obtusata*	carnivore	GCA_047404255.1	509.9 Mb
	*Sphyraena novaehollandiae*	carnivore	GCA_050436865.1	610.1 Mb
Serranidae	*Cephalopholis miniata*	carnivore		
	*Cephalopholis sonnerati*	carnivore	GCA_043388385.1	1 Gb
Lethrinidae	*Lethrinus lentjan*	carnivore		
	*Lethrinus nebulosus*	carnivore	GCA_045362495.1	1.1 Gb
	*Lethrinus variegatus*	carnivore	GCA_051253335.1	1 Gb
Lutjanidae	*Lutjanus rivulatus*	carnivore	GCA_051995215.1	1 Gb
	*Lutjanus fulviflamma*	carnivore	GCA_051362095.1	1.1 Gb
	*Lutjanus erythropterus*	carnivore	GCA_020091685.1	969.9 Mb
	*Lutjanus johnii*	carnivore	GCA_051995335.1	986.6 Mb
Siganidae	*Siganus fuscescens*	herbivore	GCA_042847805.1	498.2 Mb
	*Siganus stellatus*	herbivore		
	*Siganus canaliculatus*	herbivore	GCA_048569235.1	547.1 Mb
	*Siganus virgatus*	herbivore	GCA_051362085.1	585.4 Mb
	*Siganus vulpinus*	herbivore	GCA_051995025.1	580 Mb
	*Siganus spinus*	herbivore	GCA_049181435.1	519.2 Mb
Acanthuridae	*Acanthurus lineatus*	herbivore	GCA_029814665.1	642 Mb
	*Acanthurus tractus*	herbivore	GCA_029783725.1	712.3 Mb
	*Ctenochaetus hawaiiensis*	herbivore	GCA_029783645.1	668.1 Mb
	*Ctenochaetus striatus*	herbivore	GCA_034780835.1	674.7 Mb
	*Naso brevirostris*	herbivore	GCA_029874835.1	820.2 Mb
	*Naso vlamingii*	herbivore	GCA_051996375.1	857.5 Mb
Labridae	*Chlorurus sordidus*	herbivore		
	*Scarus ghobban*	herbivore		
	*Scarus psittacus*	herbivore		
	*Scarus rivulatus*	herbivore	GCA_051912175.1	1.6 Gb
	*Scarus forsteni*	herbivore	GCA_036419115.1	1.7 Gb
Holocentridae	*Sargocentron spiniferum*	carnivore	GCA_051994385.1	789.3 Mb
	*Myripristis murdjan*	carnivore	GCA_902150065.1	835.3 Mb
Lepisosteidae	*Lepisosteus oculatus*	outgroup	GCA_040954835.1	1.2 Gb

Genome assemblies were retrieved from NCBI, and dietary data were obtained from FishBase, and genome sizes were reported in megabases (Mb) or gigabases (Gb).

### Candidate gene selection and homology-based gene prediction

**Fig. 3:**
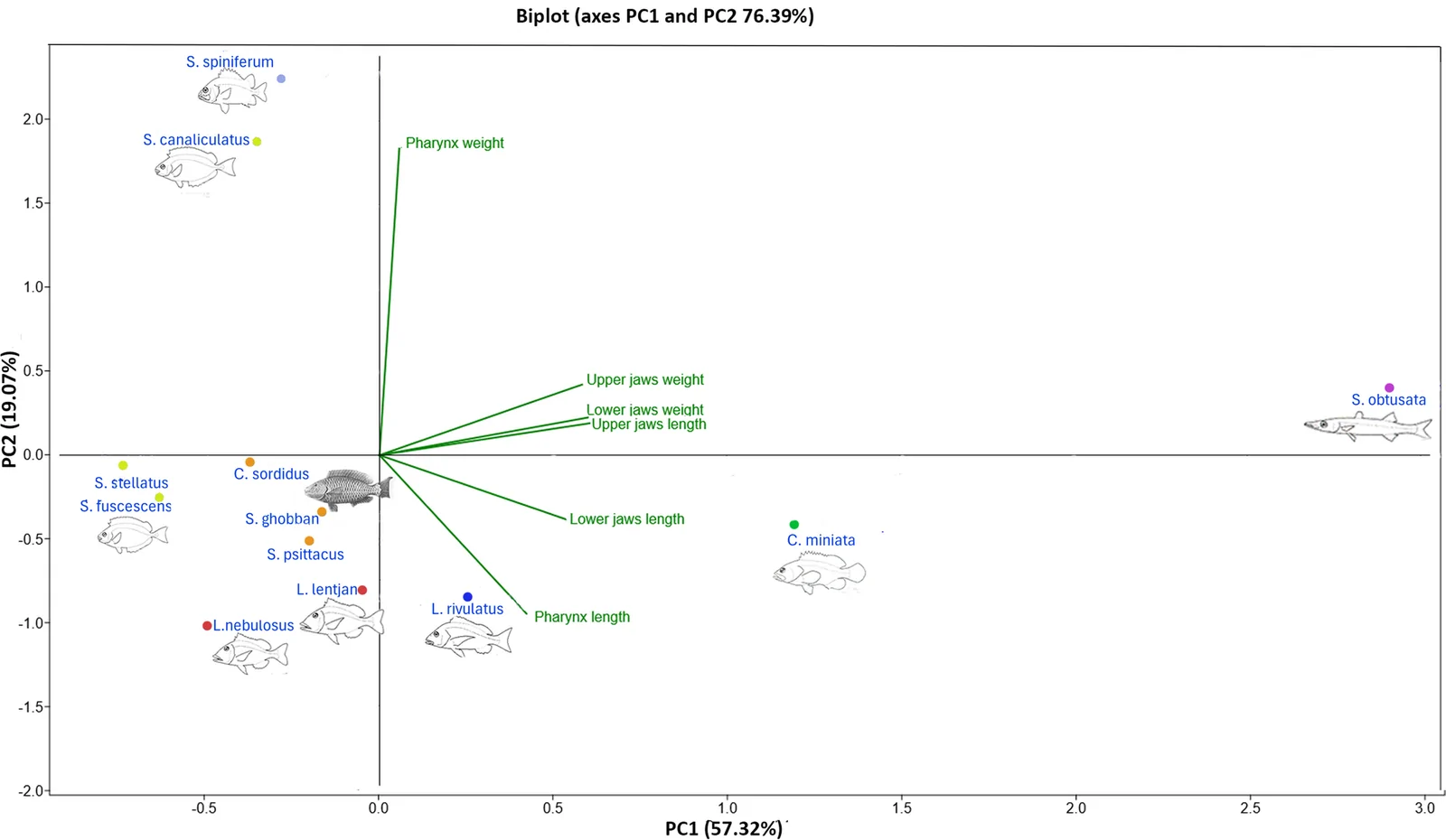
Biplot of the principal component analysis (PCA) for size-standardized morphometric measurements of fish species (lower jaw weight, upper jaw weight, pharynx weight, upper jaw length, lower jaw length, and pharynx length) in studied fish species (*S. obtusata, S. spiniferum*, *S. fuscescens, S. stellatus*, *S. canaliculatus, C. sordidus*, *S. ghobban, S. psittacus*, *C. miniata, L. rivulatus, L. lentjan*, and *L. nebulosus*)

A curated set of 130 protein-coding genes associated with tooth development, morphogenesis, and mineralization was compiled based on previously published studies (Supplementary Table S1). These genes were first identified in two well-annotated teleost reference genomes: *Danio rerio* (assembly GCA_049306965.1) and *Oryzias latipes* (assembly GCA_053564925.1). A total of 130 and 127 orthologous loci were recovered from the zebrafish and medaka genomes, respectively. Homology-based gene prediction across the target genomes was performed using Gene Model Mapper (GeMoMa v1.9) [21]. For each target genome, predictions were conducted independently using *D. rerio* and *O. latipes* as reference species. The resulting prediction sets were subsequently merged using the GeMoMa Annotation Filter (GAF) module, which selected the highest-confidence gene model per locus based on GeMoMa scoring metrics (Fig. 3). To ensure sequence integrity, only coding sequences (CDS) with lengths divisible by three and containing valid start and stop codons were retained. Following the filtering and merging, CDS along with their translated protein sequences were extracted using the GeMoMa Extractor tool. These sequences were retained for subsequent evolutionary analyses.

### Phylogenetic tree reconstruction

Protein sequences for each candidate gene were aligned across all species using MAFFT v7 [22] to ensure accurate homology detection. Poorly aligned regions were removed using trim Al v1.4 [23] to improve alignment quality and reduce phylogenetic noise. The curated alignments were then concatenated into a super matrix, and a partitioned maximum-likelihood phylogenetic analysis was performed with IQ-TREE v3 [24]. Each gene was treated as an independent partition, and the best model-fitting substitution for each partition was automatically selected using Model Finder [25]. Branch support was evaluated using 1,000 ultrafast bootstrap replicates. For species lacking complete nuclear genomic data, mitochondrial markers (COX1 and CYTB) were included to assist their placement within the phylogenetic framework. These markers were aligned independently and incorporated into the analysis to maintain taxonomic representation and accurate rooting. A concatenation-based maximum-likelihood approach was selected to verify that the selected sequences recovered the expected taxonomic relationships among the studied species and to provide a reliable phylogenetic framework for subsequent analyses. The resulting phylogeny was visualized using iTOL [26]. To investigate the evolutionary history of trophic strategies, stochastic character mapping was performed using the *make.simmap* function implemented in the R package phytools v 2.5.2. Dietary states (carnivorous or herbivorous) were mapped onto the species phylogeny using 1,000 stochastic simulations.

### Data analysis

#### Molecular selection analyses

To investigate signatures of adaptive evolution in tooth-associated genes, codon-aware selection analyses were conducted using the HyPhy framework [27]. Protein sequences for each gene were first aligned with MAFFT, and the corresponding CDS were projected onto the protein alignments using PAL2NAL [28] to generate codon-aware alignments while preserving reading-frame integrity. Poorly aligned regions were removed using trim Al to minimize potential false-positive signals in downstream analyses.

Gene-specific maximum-likelihood phylogenetic trees were inferred for each locus and served as the framework for all evolutionary tests. Gene-wide episodic positive selection was evaluated using the BUSTED model. Statistical significance was assessed using likelihood ratio tests and corrected for multiple comparisons using false discovery rate (FDR) across the 130 tested genes. Genes that remained significant after FDR correction (FDR < 0.05) were further analyzed using a BSREL to detect branch-level episodic selection. Foreground branches labeled according to dietary classification (herbivore or carnivore), and the proportion of branches exhibiting significant selection was quantified separately for each dietary group.

Complementary analyses were conducted to characterize shifts in selective regimes. RELAX was applied to detect shifts in selection intensity, identifying branches under relaxed or intensified selection, while MEME was used to detect site-level episodic selection. These complementary analyses were reported separately to clearly distinguish gene-wide, branch-specific, and site-level selection patterns.

#### Gene enrichment and structural analysis

Functional enrichment analysis was performed using ClusterProfiler [29] to identify significant biological functions and pathways associated with the candidate genes. To further explore their structural features, three-dimensional protein structures were predicted using AlphaFold 3 [30]. Potential active sites within the predicted structures were predicted using P2Rank [31]. These analyses provided complementary functional and structural insights into the candidate proteins.

#### Statistical analysis of the morphometric data of fish species

Morphometric and phenotypic data of the studied fish species are presented as mean ± standard deviation (SD) of three replicates (*n* = 3). Before performing the analysis, the normality and homogeneity of variances of the data were verified using the Shapiro-Wilk and Levene’s tests, respectively. Subsequently, morphometric characters were analyzed using one-way analysis of variance (ANOVA) to evaluate phenotypic differences among species [32, 33]. When significant differences were detected, Tukey’s multiple comparisons test was used to determine pairwise differences between groups. A *p*-values < 0.05 were considered statistically significant. Data processing and statistical analysis were carried out with Minitab version 19 and PAST version 4.03 [34]. Means that did not share a common letter were significantly different across tables and figures. To eliminate the confounding effect of individual body size variation on both linear and weight measurements, all morphometric characters were standardized using the allometric scaling method proposed by [35]. The standardized values M_std_ were calculated using the following formula: M_std_ = M_i_ (L_0_/L_i_)^b^

where: M_i_ is the original morphometric measurement, L_i_ is the standard length of the individual fish, L_0_ is the overall mean standard length of all sampled specimens, and b is the allometric growth coefficient derived from the linear regression of log (M_i_) on log (L_i_). Pearson correlation coefficients were calculated among the major size-standardized morphometric variables (including lower jaw weight, upper jaw weight, pharynx weight, upper jaw length, lower jaw length, and pharynx length), using GraphPad Prism version 10.4, and principal component analysis (PCA) was performed with XLSTAT to characterize morphological variation among species. All multivariate analyses, including Principal Component Analysis (PCA) and Pearson correlation, were performed using the size-standardized dataset to ensure that the observed patterns represent functional morphological variations independent of individual body size.

### Ethical statements

This study was conducted in accordance with Egyptian laws and the guidelines of the Faculty of Science, Committee on Animal Welfare and Ethics, Tanta University (Approval number: IACUC-SCI-TU-0511).

## Results

### Gross anatomy and morphometric findings

**Fig. 4:**
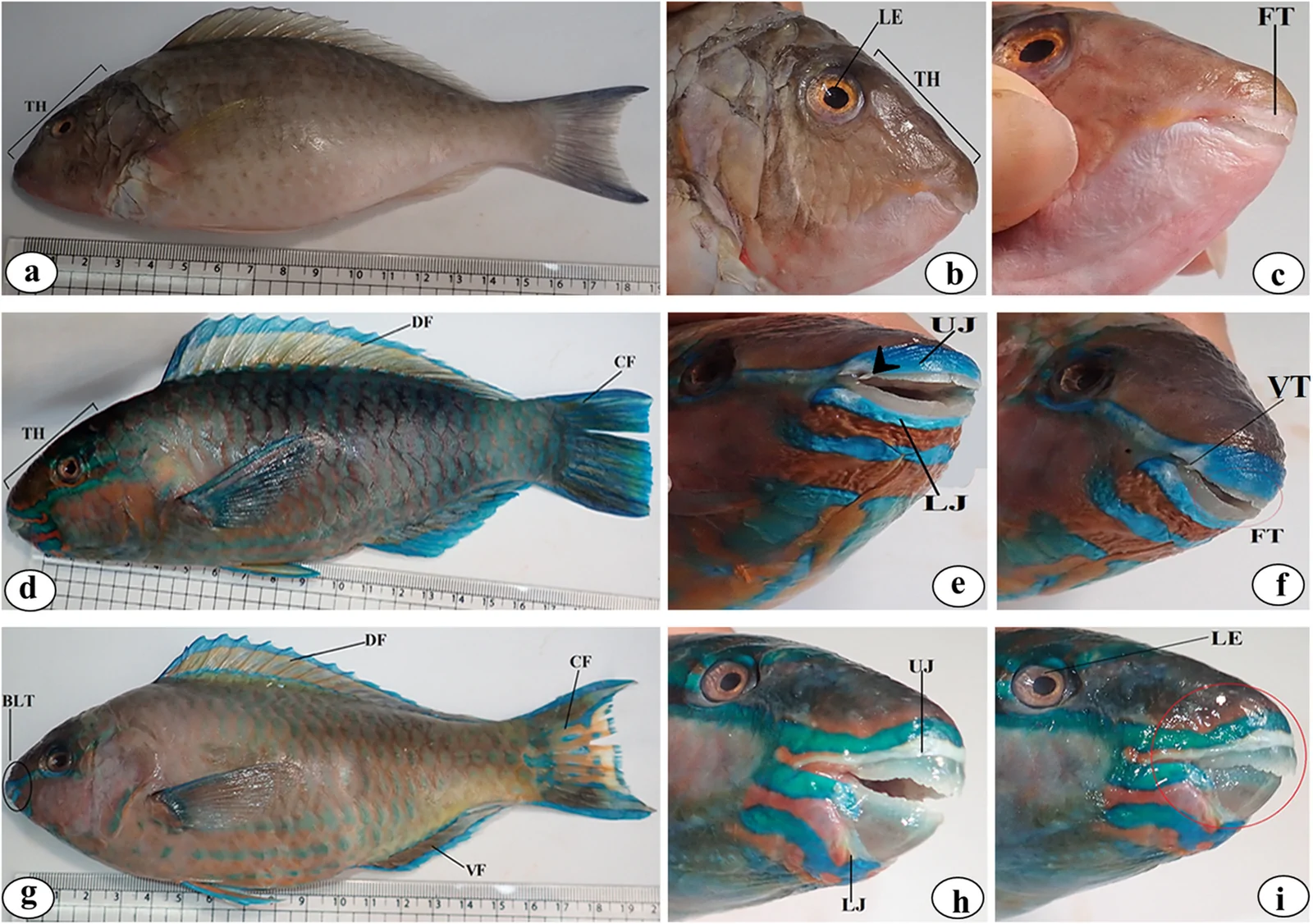
(**a-i**) Photographs of species the family Scaridae; (**a-c**) C. sordidus characterized by distinctive and rounded head shape (resembling a bullet) (TH). (**d-f**) S. ghobban characterized by blue-edged fins, especially dorsal and tail. (**g-i**) S. psittacus characterized by iridescent scales and striped or blotchy facial markings, thick, oval-shaped body with large and visible scales had bright blue-green coloration with pink, purple, or reddish markings. The thick head in this family contains large eye (LE) and the mouth protrudes downward giving the typical beak shape by fused teeth (FT). Tail fin is straight or slightly curved (CF). Tightly packed and fused teeth together (FT) forming a serrated denticulate strong parrot beak-like structure made of two dental plates (one upper (UJ), one lower (LJ)). Note: the presence of upper one pair of sharp prominent villiform lateral teeth in S. ghobban (**VT,** **black head arrow**)

**Fig. 5:**
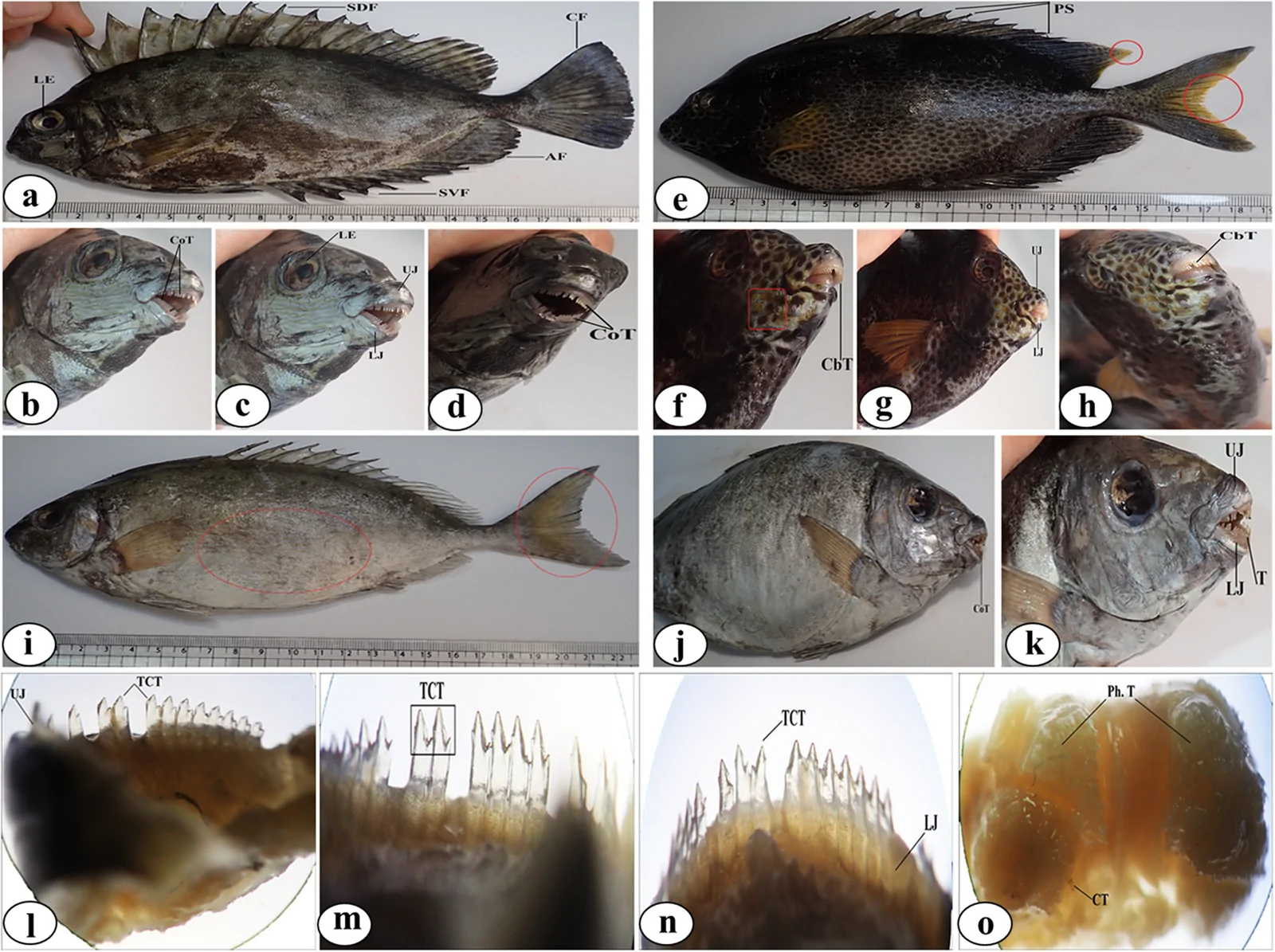
(**a-k**) Photographs of species of the family Siganidae; (**a**) S. fuscescens appears oval, laterally compressed with mottled brown to gray with darker patches or marbling, it had large eyes (LE), dorsal fin with strong 13 spines (SDF); these spines were venomous in dorsal, anal (AF), spiny ventral (SVF), and pelvic fins. (**b-d**) Small mouth with herbivorous comb teeth (CoT). (**e-h**) S. stellatus with a spotted or mottled pattern appears dark laterally compressed with noticeable black spots or blotches (**red rectangle**). The caudal fin and edges of dorsal and anal fins show a yellowish hue (**red circle**). (**i-k**) S. canaliculatus appears slightly elongated and laterally compressed with small head and large eye, it has grayish-silver coloration with an olive tint, featuring small pale spots. The dorsal fin contains prominent spines and a clearly forked tail (**red circle**). (**l-o**) Photomicrographs of jaws with their teeth; (**l-m**) upper jaw (UJ) with tricuspid teeth (TCT). (**n**) lower jaw (LJ) with enlarged tricuspid teeth (TCT). (o) Pharyngeal teeth arranged in four pads and appeared mainly conical (CT)

The Bullet head Parrotfish *(C. sordidus),* Blue-barred Parrotfish *(S. ghobban),* and Common Parrotfish *(S. psittacus)* belonged to the herbivorous family Scaridae. Their average length and weight were 20.66 ± 1.52 cm and 109.66 ± 5.5 g for *C. sordidus*, 21.66 ± 1.53 cm and 158.33 ± 7.76 g for *S. ghobban*, and 21.33 ± 1.53 cm and 149.66 ± 2.08 g for *S. psittacus*, respectively (Figs. 1and 4). These species were characterized by thick, oval bodies with large, bright scales showing blue-green coloration with pink, purple, or reddish markings. The head was thick with large eyes, and the mouth protrudes downward forming beak-like structure due to fused teeth. The caudal fin was straight or slightly curved (Fig. 5a–i). *C. sordidus* had a unique, rounded (bullet-like) head shape (Fig. 5a–c), *S. ghobban* showed blue-edged fins, especially on the dorsal and caudal fins (Fig. 5d–f), while *S. psittacus* characterized by iridescent scales and striped or blotchy facial markings (Fig. 5g–i).

**Fig. 6:**
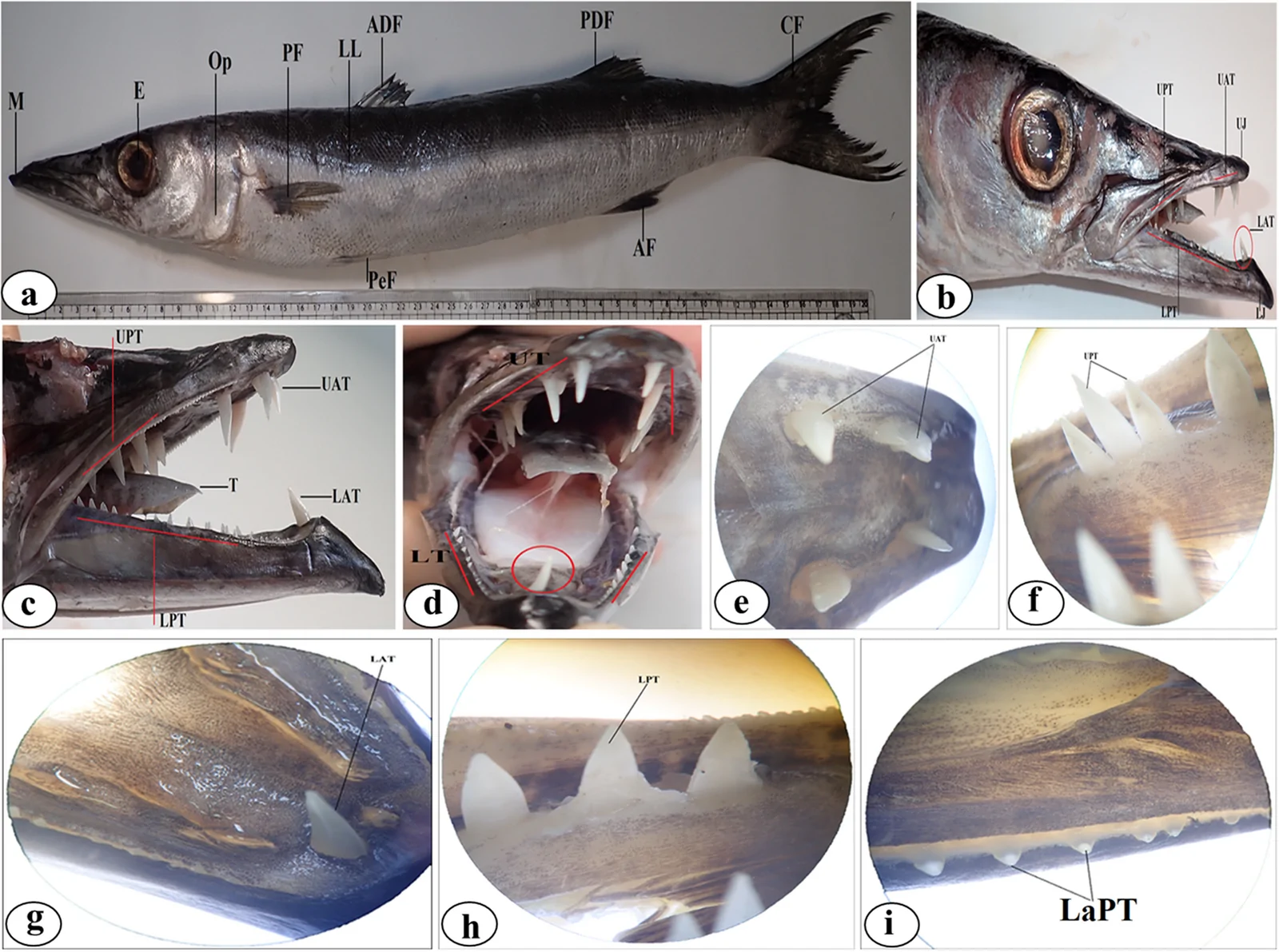
(**a-d**) Photographs of S. obtusata showing: (**a**) whole body has an elongate and cylindrical shape with a silver coloration and a darker back with quite large pointed head has prominent eyes (**E**), large protractile mouth (M). Widely separated dorsal fins (anterior (ADF) and posterior (PDF)), pectoral fin (PF), pelvic fin (PeF), anal fin (AF), and caudal fin (CF). (**b-d**) Large wide protractile mouth with a distinctly protruding lower jaw (LJ) which juts out beyond the upper one (UJ), it has large long sharp- edged fang like pointed conical teeth arranged into; upper anterior (UAT) and lower anterior strong canine (LAT). bilateral pairs of large strong canine teeth were lined the tip of premaxilla, they were recurved fang-like protrude from the symphysis that connects the two bilateral elements and fits neatly in a recess between the anterior most canines of the upper jaws; upper posterior (UPT), and lower posterior (LPT). (**e-i**) Photomicrographs of teeth on jaws showing: (e) Four upper anterior teeth (UAT). (**f**) Single median lower anterior tooth found on the midline of the lower jaw (LAT). (**g**) Upper posterior teeth (UPT). (**h**) Lower posterior (LPT). (**i**) Lateral posterior teeth (LaPT) appeared as serrated short, broad, straight with truncate edge impaling the maxilla

The family Siganidae (rabbit-fishes) includes herbivorous species that feed on macro algae and sea grasses, and occasionally small invertebrates. It comprises Dusky rabbit-fish (*S. fuscescens*), Brown spotted rabbit-fish *(S. stellatus),* and White-spotted Spine foot (*S. canaliculatus)*, these species inhabited shallow coastal reefs, lagoons, and sea-grass beds in the Red Sea and Indian Ocean at depth 1 to 30 m (Fig. 1). *S. fuscescens* had an oval, laterally compressed body with a mottled brown to gray with darker spots or marbling (Fig. 6a). Its average length was 20.33 ± 1.52 cm, and its average weight was 149.33 ± 6.5 g (Fig. 4). It had large eyes, a small mouth adapted for herbivory, and a dorsal fin with thirteen strong spines; the dorsal, pelvic, and anal spines were venomous (Fig. 6b–d). *S. stellatus* had an average length of 20.66 ± 1.52 cm and weight of 109.33 ± 10.4 g (Fig. 4). Its body was laterally compressed with dark spots or blotches, and the caudal fin and fin margins showed a yellowish hue (Fig. 6e–h). *S. canaliculatus* was slightly elongated and laterally compressed, with a grayish-silver body and olive tint with small whitish patches. Its average length was 23 ± 1.73 cm and weight 100.33 ± 4.04 g (Fig. 3). It had a small head, large eyes, a forked caudal fin, and prominent dorsal spines (Fig. 6i–k).

**Fig. 7:**
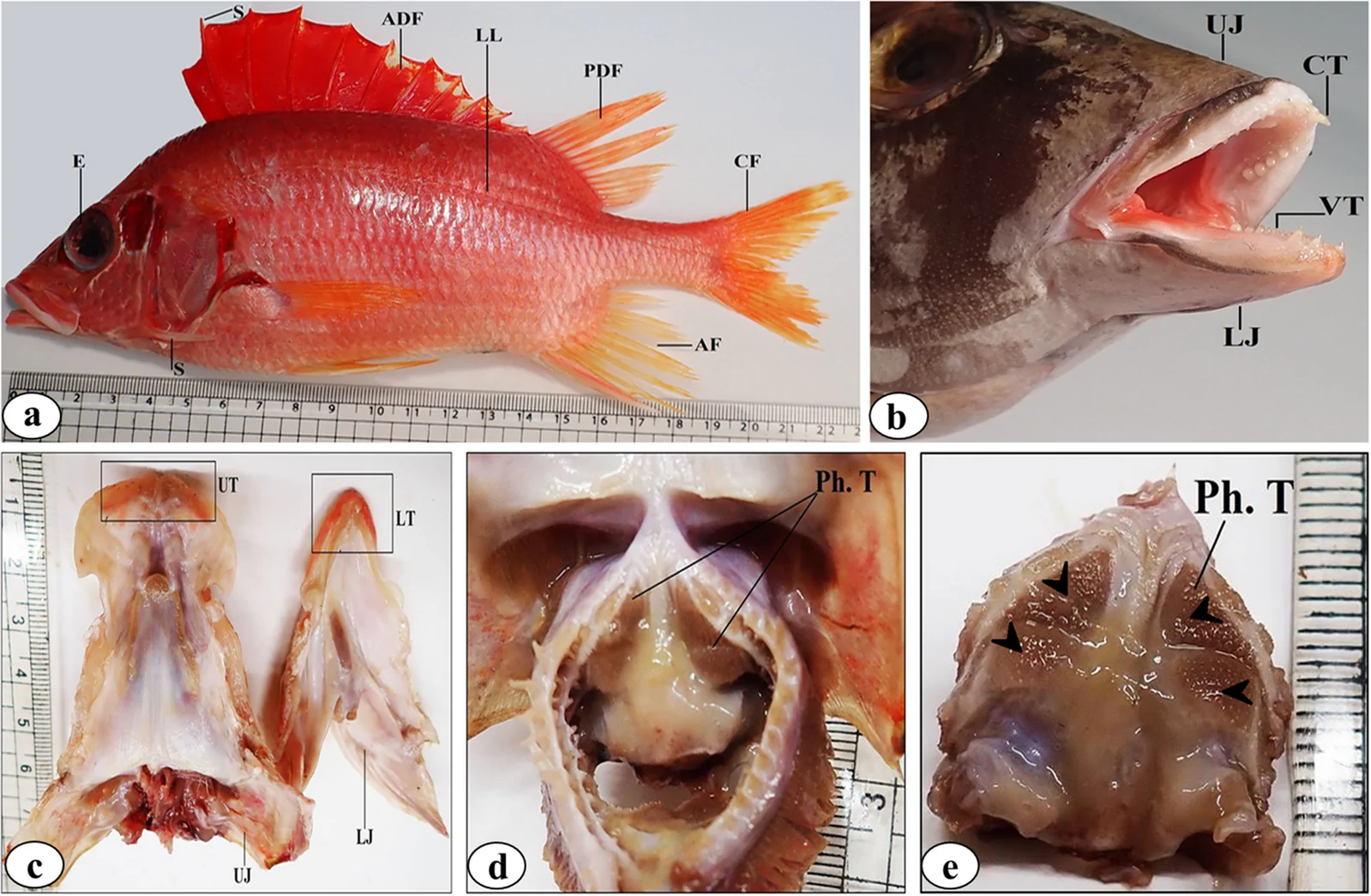
(**a-e**) Photographs of S. spiniferum showing: (**a**) deep and compressed bright body with red coloration and covered with rough, ctenoid large and reflective scales. It has very large eye (**E**), and operculum supported by strong spines (**S**). Orange-yellowish fins were differentiated as composed of (anterior dorsal (ADF) and posterior dorsal (PDF)), caudal (CF), and anal (AF). (**b**) Large mouth has three types of teeth; prominent anterior canine teeth (CT), small sharp and conical teeth were arranged in multiple rows along both the upper (UJ) and lower jaws (LJ), and villiform small and slender teeth (VT) also present on the vomer in the form of V-shaped patch of vomerine teeth and palatine which were closely set in dense bands or patches, resembling the bristles of a brush. (**c**) Small teeth arranged in bands on the strong long jaws and on the roof of the mouth (palatines); (UT) and (LT). (**d, e**) Pharyngeal teeth (Ph. T) compose of two halfs and arranged into four pads (**black head arrow**)

The family Sphyraenidae includes carnivorous obtuse barracuda (*S. obtusata*), a predatory species found in marine and brackish water at depths of 5–200 m in the Indo-Pacific, including the Red Sea (Fig. 1). Its average length was 53 ± 1.73 cm and weighed 699.66 ± 10.01 g (Fig. 4). The body was cylindrical and elongated with a darker dorsal side and silver coloration. The dorsal fins were widely separated (Fig. 7a). The head was large with prominent eyes, and the lower jaw protrudes beyond the upper jaw, forming a large, pointed, pick-like, and protractile mouth (Fig. 7b, c).

**Fig. 8:**
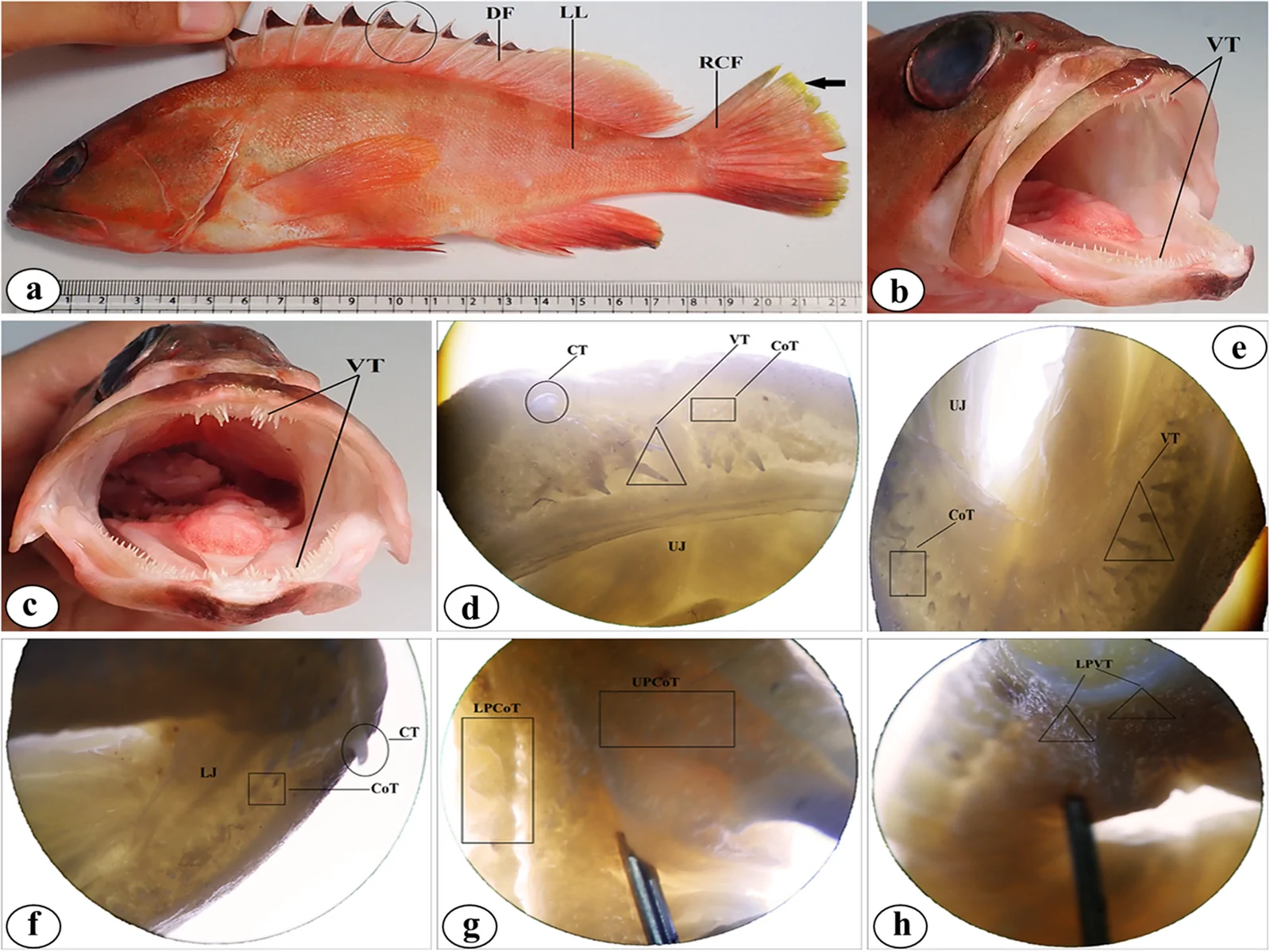
(**a-h**) Photographs of C. miniata: (**a-c**) body characterized by robust compressed with vibrant reddish or orange coloration with scattered small blue spots and a large mouth with multiple rows of teeth (VT). It had well-developed spiny dorsal fins (DF) and rounded caudal fin (RCF). (**d-f**) Photomicrographs of upper (UJ) and lower (LJ) jaws with multiple rows of teeth; the anterior canine (CT), conical (CoT), and villiform (VT). (**g-h**) Photomicrographs of pharyngeal teeth have three types; upper conical (UPCoT), lateral conical (LPCoT), and lower (LPVT)

The family Holocentridae includes the carnivorous Sabre squirrelfish (*S. spiniferum*), which inhabits tropical coral reef in the Indo-Pacific region at depths of 2–40 m (Fig. 1). Its average length was 22 ± 1.01 cm and weight 201.66 ± 11.67 g (Fig. 4). It had a deep, compressed red body with large, reflective ctenoid scales and orange-yellowish spiny fins, particularly on the dorsal side. The eyes were large, indicating nocturnal adaptation (Fig. 8a, b).

**Fig. 9:**
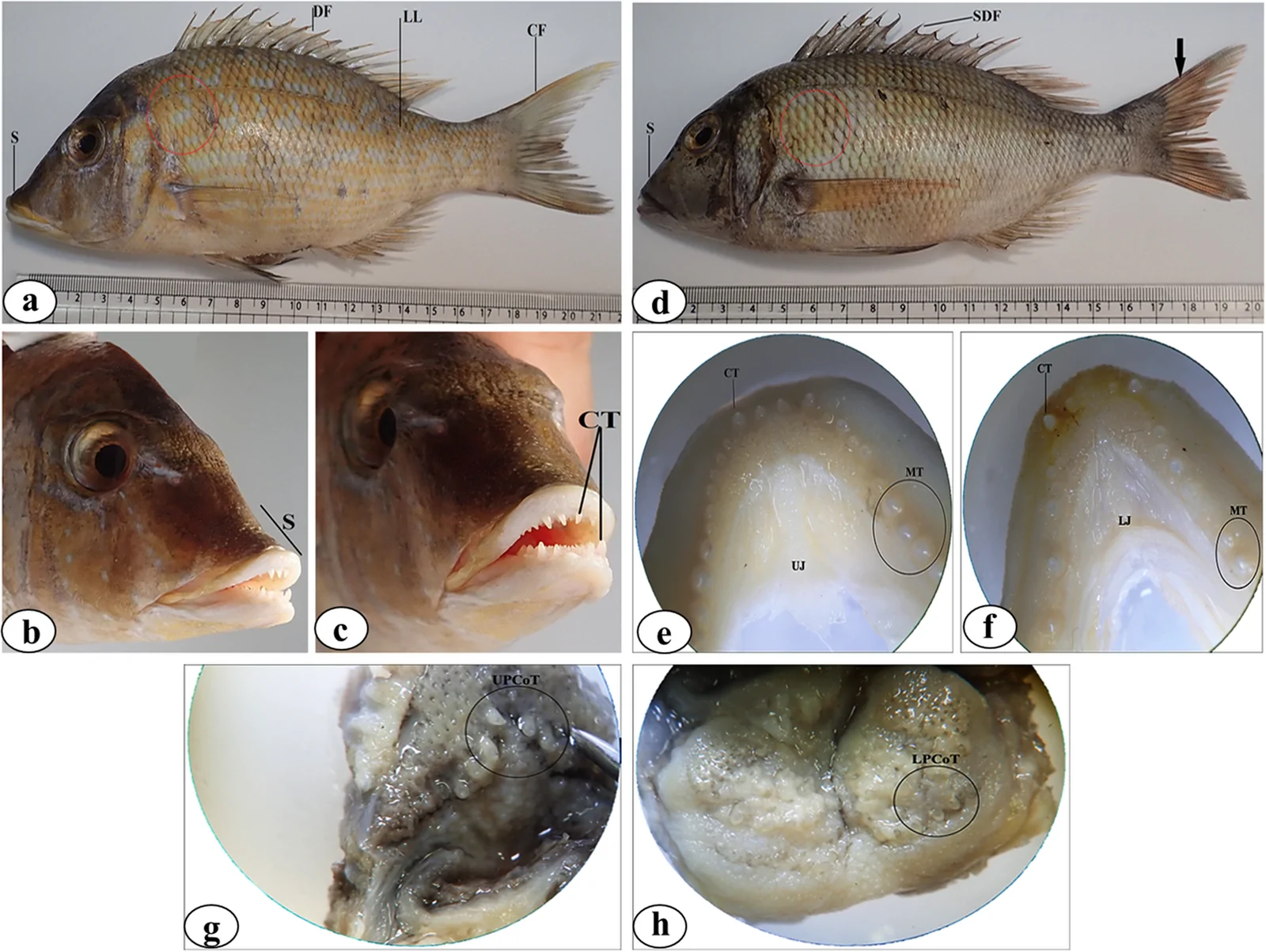
(**a-d**) Photographs of the species of family Lethrinidae: (**a-c**) L. lentjan has oblong and moderately deep-bodied with light brown to greyish body with yellowish to bluish scale patterns. It had long dorsal fin (DF) with distinct spines (about 10) followed by soft rays and slightly forked tail fin (CF). Prominent snout slightly pointed mouth (**S**) has conical canine teeth in front (CT). (**d**) L. nebulosus has elongated body with a slightly forked tail (**black arrow**) with brownish or greyish with faint blotches or stripes and prominent spiny dorsal fin with sharp tips (SDF). Pointed snout with a strong jaw (**S**). (**e-f**) Photomicrographs of upper (UJ) and lower jaws (LJ) have conical canine teeth in front (CT) and molariform ones (MT) posteriorly. (**g, h**) Photomicrographs of upper (UPCoT) and lower pharyngeal conical teeth (LPCoT)

The family Serranidae includes the carnivorous Coral hind grouper (*C. miniata*), widely distributed in the Red Sea, Indian Ocean, and Western Pacific at depths of 2–150 m (Fig. 1). Its average length was 23.33 ± 0.57 cm and weight 171.33 ± 3.05 g (Fig. 4). It has robust, compressed body, well-developed spiny dorsal fins, a rounded caudal fin, and a reddish or orange coloration with sporadic tiny blue dots are its defining characteristics (Fig. 9a–c).

**Fig. 10:**
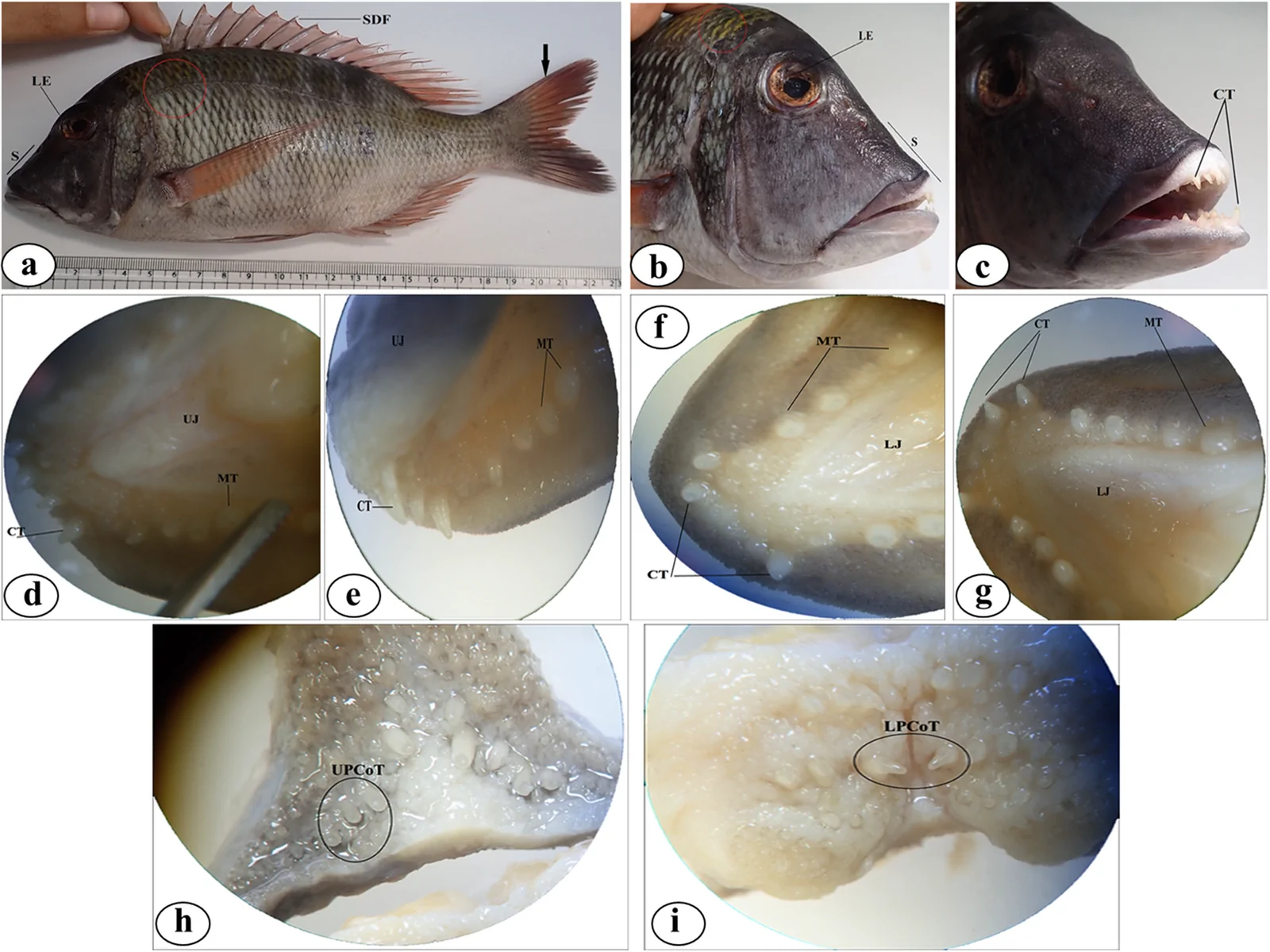
(**a-c**) Photographs of elongated and moderately compressed L. rivulatus has grayish to silvery body with a pinkish hue; reddish fins, especially the dorsal (SDF), anal, and caudal fins (**black arrow**). A reddish-pink patch or spot near the operculum (gill cover)-the “pink ear” (**red circle**), and snout relatively blunt (**S**). (**d-g**) Photomicrographs of teeth on upper and lower jaws; canine teeth (CT) anteriorly and molariform ones (MT) backwards. (**h-i**) Photomicrographs of conical teeth on upper and lower jaws (UPCoT and LPCoT)

**Fig. 11:**
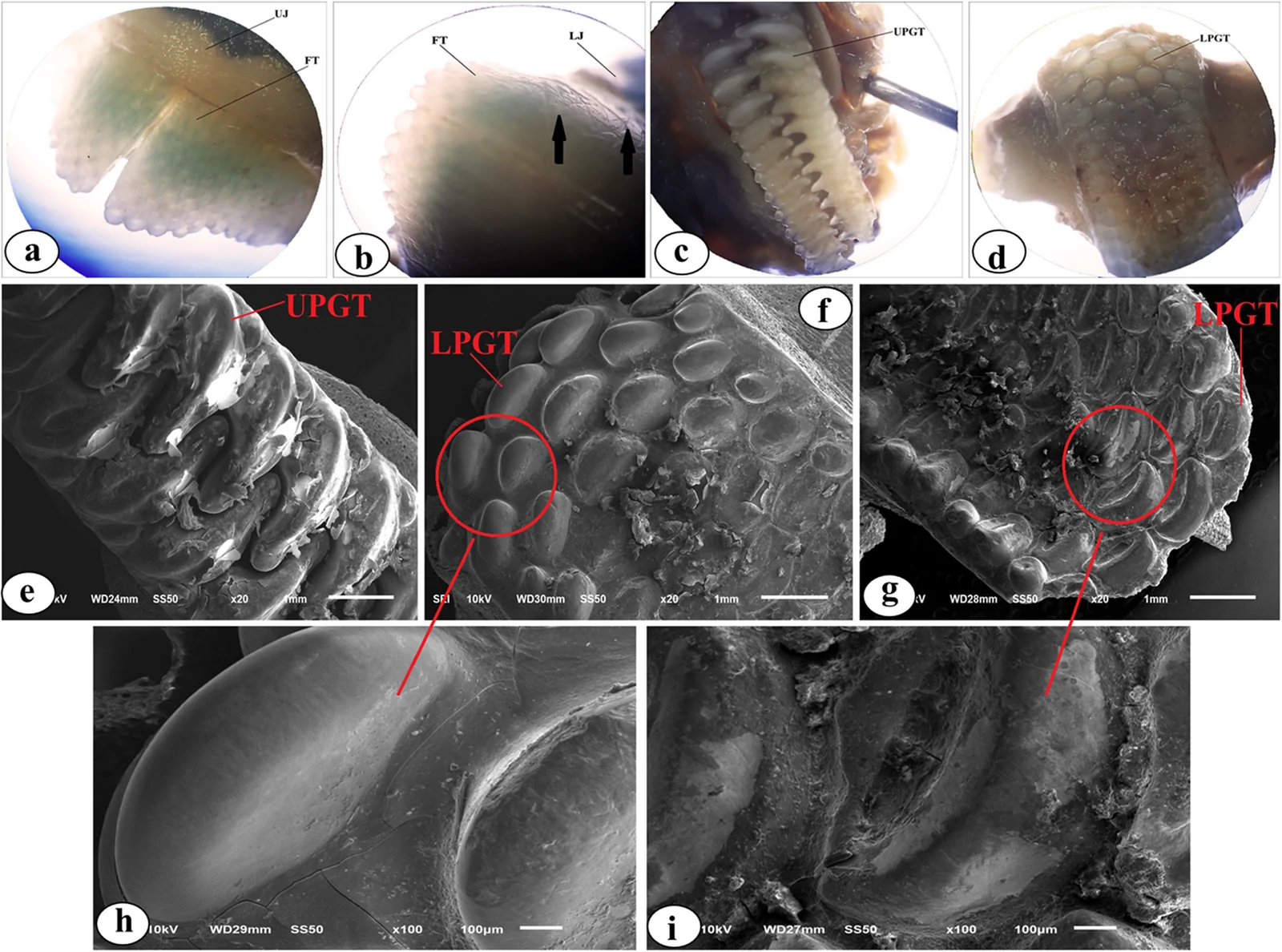
(**a-b**) Photomicrographs of tightly packed and fused teeth together (FT) forming a serrated denticulate strong parrot beak-like structure made of two dental plates (one upper (UJ), one lower (LJ)). Note: the presence of small pits or grooves found on the beak surface (**black arrow**). (**c-d**) Photomicrographs of upper (UPGT) and lower (LPGT) pharyngeal teeth. (**e-i**) Scanning electron micrographs of pharyngeal teeth of S. ghobban showing: hard chewing pads (one upper (UPGT) and one lower (LPGT)) found inside the throat, composed of fluorapatite crystals which were arranged in bundles, many oriented perpendicularly or vertically to the enameloid surface and it has stiff hard grinding teeth (pharyngeal mill)

The family Lethrinidae includes Pink ear emperor (*L. lentjan)* and Blubber lip snapper *(L. nebulosus)*, while the family Lutjanidae (Snappers) includes Blotched snapper (*L. rivulatus*). These species inhabited coral reefs, sandy bottoms, and sea-grass beds in the Indo-Pacific, including the Red Sea (Fig. 1). *L. lentjan* had an average length of 20.66 ± 1.52 cm and weight of 141.33 ± 4.93 g and *L. nebulosus* had an average length of 21.33 ± 1.53 cm and weight of 113.66 ± 4.5 g (Fig. 4). *L. lentjan* had an oblong, relatively deep-bodied with a light brown to grey coloration and yellowish to bluish scale patterns, a long dorsal fin with about ten conspicuous spines, and a slightly forked caudal fin (Fig. 10a–c). *L. nebulosus* was characterized by a pointed snout, strong jaw, spiny dorsal fin, and a brownish body with faint markings (Fig. 10d). *L. rivulatus* had an average length of 23.33 ± 1.52 cm and weight of 259.33 ± 8.08 g (Fig. 4). It had an elongated and slightly compressed body with a grayish-silvery color and pinkish hue, reddish fins, and characteristic pink patches close to the operculum (pink ear), and the snout was comparatively blunt (Fig. 11a–c). The L. lentjan and L.nebulosus exhibited distinct tooth types: anterior prominent canines that were conical for gripping, and molariform or rounded vomerine teeth in other posterior regions for crushing or processing hard-shelled organisms as mollusks and crustaceans (Fig. 10e, f). This dentition type is characteristic of many emperor fish species that adapted for grabbing or grasping, piercing, and holding prey. The dentition of the snapper L. rivulatus included conical canine-like teeth for catching prey and blunt posterior teeth or edges for processing prey (Fig. 11d–g). Conical and sharp, pharyngeal teeth were designed to grasp and manipulate prey like fish, crabs, and cephalopods. Molariforms, which were broad, blunt, and flattened, have evolved to crush, grind, and decompose hard-shelled prey, such as crabs and mollusks (Fig. 10g, h and  11h, i).

### Principal component analysis of morphometric measurements of fish species

To elucidate the phenotypic differentiation among the twelve studied fish species independent of body size, a Principal Component Analysis (PCA) was performed on the size-standardized morphometric dataset. The first two principal components (PC1 and PC2) combined to explain 76.39% of the total morphological variance (Fig. 4).

PC1 accounted for the majority of the variation (57.32%) and was primarily defined by variables associated with the oral jaw apparatus. Positive loadings along this axis were strongly driven by upper jaws weight, lower jaws weight, upper jaws length, and lower jaws length. The piscivorous apex predator, *S. obtusata*, was segregated at the extreme positive end of PC1, reflecting a robust oral jaw morphology optimized for high biting force and prey capture. Conversely, species characterized by specialized, low mass grazing apparatuses, namely *S. fuscescens*, *S. stellatus*, *C. sordidus*, *S. ghobban*, and *S. psittacus*, clustered within the negative region of PC1, indicating a minimized mechanical investment in oral jaw dimensions.

PC2 explained an additional 19.07% of the variance, capturing a functional trade-off in the pharyngeal processing apparatus. This axis was characterized by a strong positive correlation for Pharynx weight and a negative correlation for Pharynx length. The durophagous predator *S. spiniferum* exhibited a pronounced positive loading along PC2 (accompanied also by high PC2 values in *S. canaliculatus*), aligning directly with the Pharynx weight vector, which underlines its specialized hypertrophy for crushing hard shelled prey. In contrast, invertivores and carnivores such as the emperor fishes (*L. lentjan* and *L. nebulosus*) and *L. rivulatus* shifted toward the negative region of PC2, corresponding with elongated pharyngeal structures (Pharynx length). *C. miniata* occupied a distinct position in the lower right quadrant, combining positive PC1 loadings with negative PC2 scores, closely aligned with the lower jaw length and Pharynx length vectors. The parrotfishes *S. psittacus* and *S. ghobban* occupied a intermediate to negative position along both axes, balancing moderate pharyngeal length with specialized herbivorous feeding mechanisms.

Taken together, these size-independent ecomorphological patterns provide compelling empirical evidence of functional niche partitioning, thereby facilitating co-existence and minimizing interspecific competition within the Marsa Alam coral reef ecosystem.

**Fig. 12:**
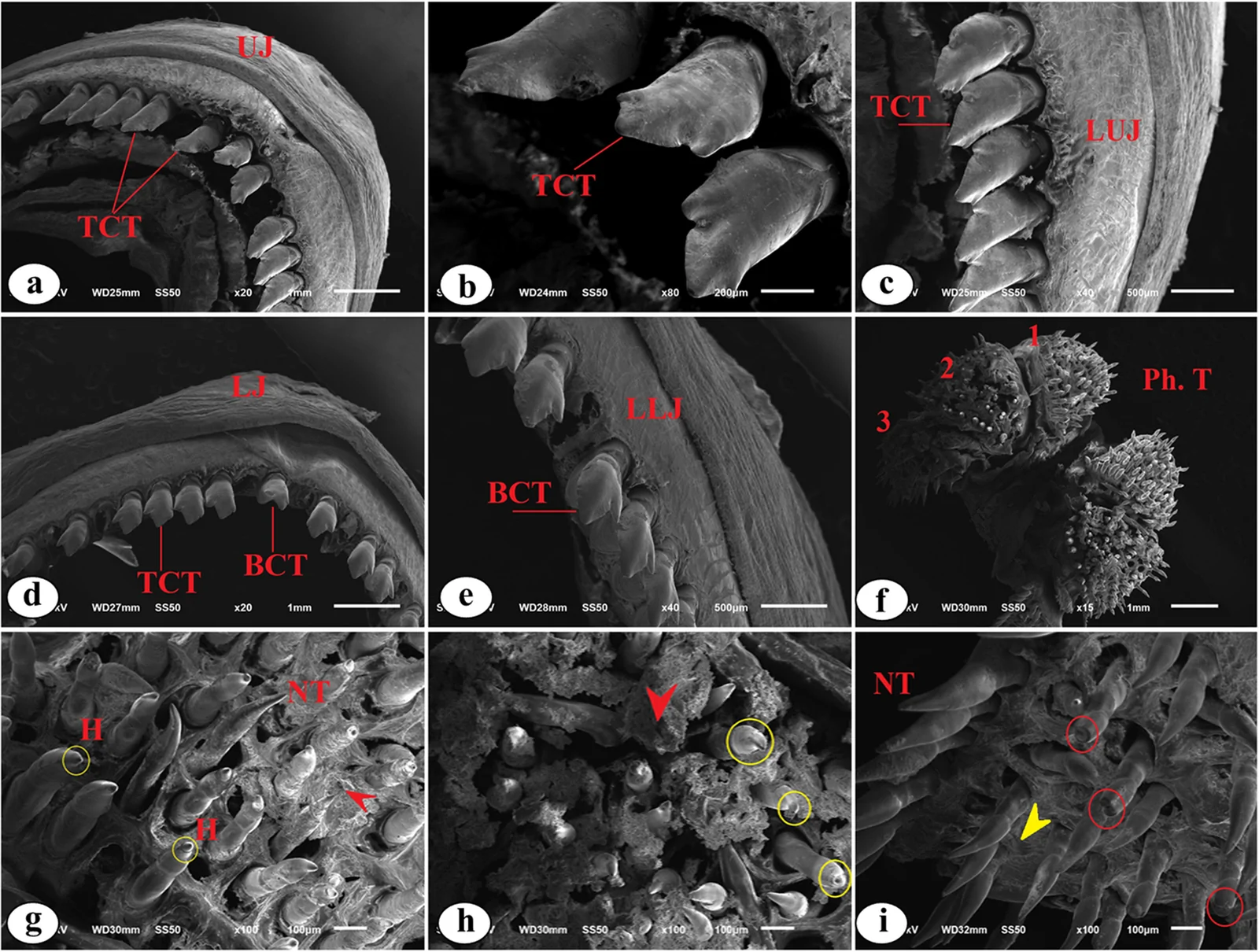
(**a-i**) Scanning electron micrographs of teeth in family Siganidae showing: (**a-e**) two types of teeth which arranged in a single row in the mouth: tri-cusped (TCT) and bi-cusped (BCT) on upper (UJ) and lower (LJ) jaws. The small tricuspid teeth were closely packed and looked like combs or small plates (CoT). (**f-i**) scanning electron micrographs of the pharyngeal teeth (Ph. T) of the roof of the buccal cavity showing: (**f**) two halfs on each side of the mid line; each half has three parts or pads; upper (1), middle (2), and lower (3). (**g-i**) some needle like teeth (NT), others carry a hook like process (H, yellow circle). Microridges and protuberances at the base surface (**yellow head arrow**), and taste bud (TB, red circle)

The Pearson correlation heatmap computed for the six size-standardized morphometric variables further elucidates the intrinsic structural relationships independent of total body size (Fig. 12). The matrix revealed a highly pronounced morphological integration within the oral jaw apparatus. Specifically, a very strong and statistically robust positive correlation was observed between upper jaws weight and lower jaws weight (*r* = 0.94), coupled with a strong positive association between their respective lengths (*r* = 0.82). Furthermore, moderate-to-strong localized integrations were noted between the mass and linear dimensions of the jaws, such as the correlation between upper jaws weight and upper jaws length (*r* = 0.71), and lower jaws weight and lower jaws length (*r* = 0.52).

Conversely, the pharyngeal processing apparatus exhibited a distinct functional decoupling from the oral jaw mechanism. Pharynx weight displayed negligible to weak correlations with both lower jaws weight (*r* = 0.08) and upper jaws weight (*r* = 0.12). Within the pharyngeal unit itself, an underlying biomechanical trade-off was identified, as evidenced by the negative correlation between Pharynx length and Pharynx weight (*r* = −0.16). Additionally, Pharynx weight showed a minor negative trend with lower jaws length (*r* = −0.04).

These distinct, compartmentalized correlation patterns demonstrate that once the confounding effects of individual body size are statistically eliminated via allometric scaling, the trophic morphology of the studied species is organized into two autonomous functional modules: the oral jaw apparatus for prey capture and the pharyngeal apparatus for prey processing.

### Stereoscopic and scanning electron microscopic investigations

**Fig. 13:**
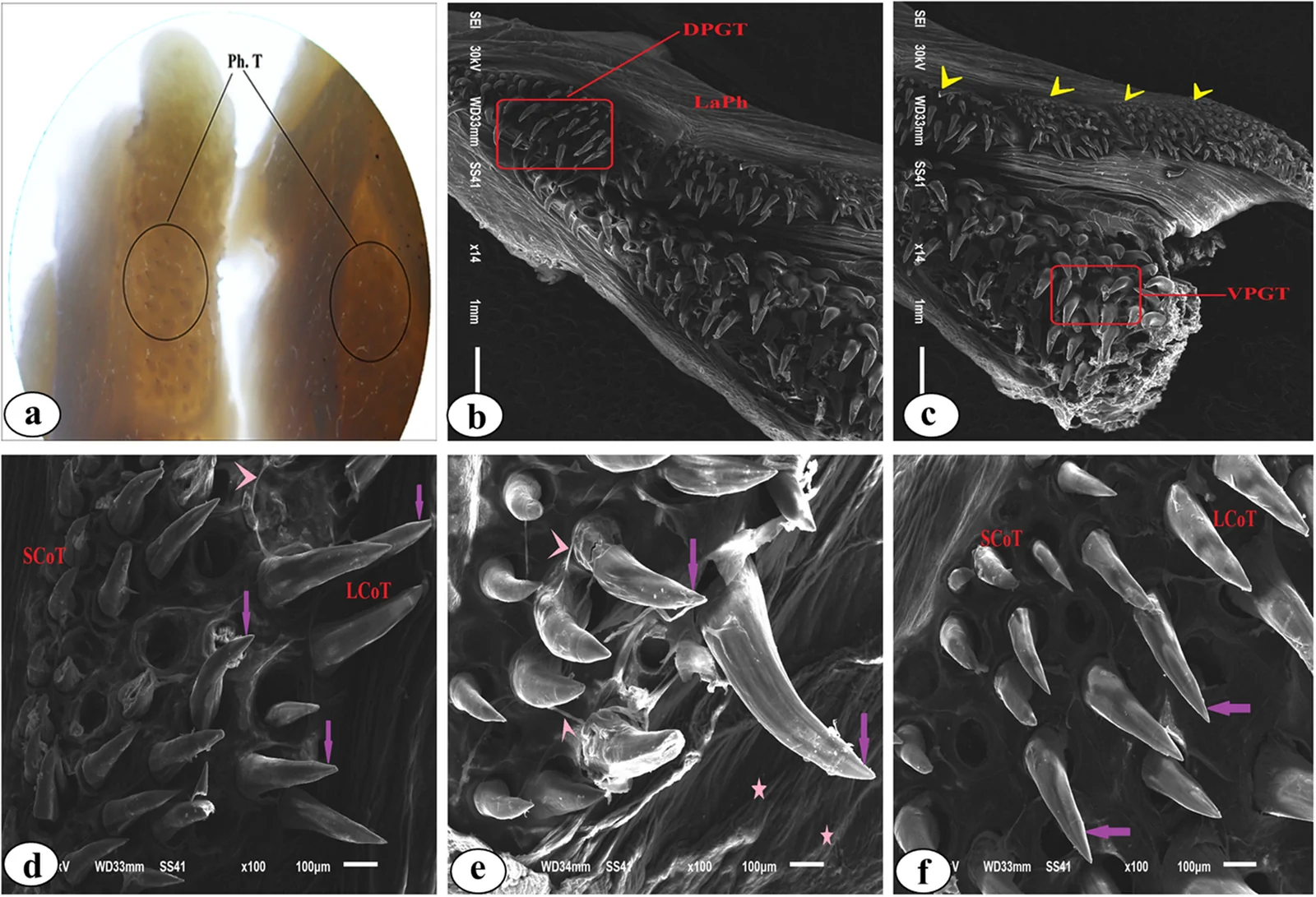
(**a**) A photograph of two halfs of S. obtusata pharyngeal teeth (Ph. T). (**b-c**) Scanning electron micrographs of S. obtusata dorsal (DPGT) and ventral (VPGT) pharyngeal conical teeth arranged in groups (**yellow head arrows**). (**d-f**) Conical teeth appeared either small (SCoT) or long (LCoT) with smooth enamel surfaces had microserrations found on very sharp cutting edges (violet arrows). Note: shallow folds on the pharyngeal surface (pink stars) and presence of ligament (pink head arrow) at the base of conical teeth to anchor them together

The parrotfishes *C. sordidus*, *S. ghobban*, and *S. psittacus* showed closely packed, fused teeth forming upper and lower dental plates (Fig. 13a). Sharp lateral villiform teeth were observed along the margins of the beak structure (Fig. 13a). Micro-topographical analysis of the beak surface revealed the presence of distinct small pits and micro-grooves (Fig. 13b). Concurrently, scanning electron microscopy of the pharyngeal teeth (pharyngeal mill) showed highly ordered bundles of fluorapatite crystals (Fig. 13c–i).

**Fig. 14:**
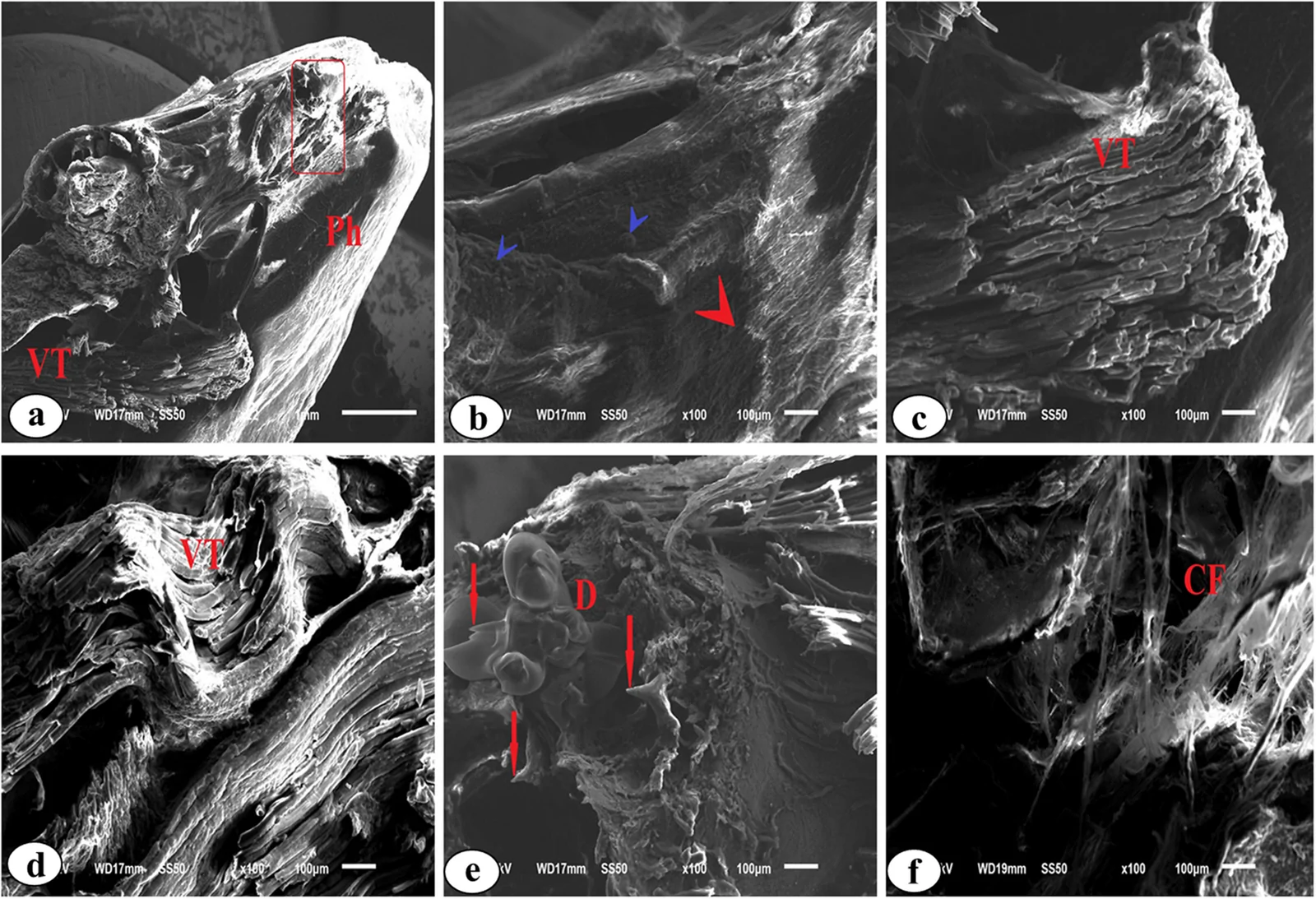
(**a-f**) Scanning electron micrographs of S. obtusata lateral pharyngeal teeth showing: (**a**) Pharyngeal surface (Ph) has villiform teeth (VT) and fibers (**red rectangle**) act as ligament to provide mechanical support and anchor the pharyngeal teeth. (**b**) Smooth pharyngeal surface (red head arrow) with mucous secretions (blue head arrows). (**c, d**) Smooth enamel surfaces of villiform teeth (VT) with small ridges or microgrooves, especially on cutting edges, to help with prey grip. (**e**) Layers of enameloid over dentin (**D**), possibly crystalline structure differences, lines of incremental growth, or microfractures. Note: microserrations or very sharp cutting edges were found on the teeth edges (blue arrows), and small, sharp, and conical pharyngeal teeth appear as spines (**red arrow**). (**f**) Fibrous connective had a dense mesh of interlacing collagen fibers (CF)

**Fig. 15:**
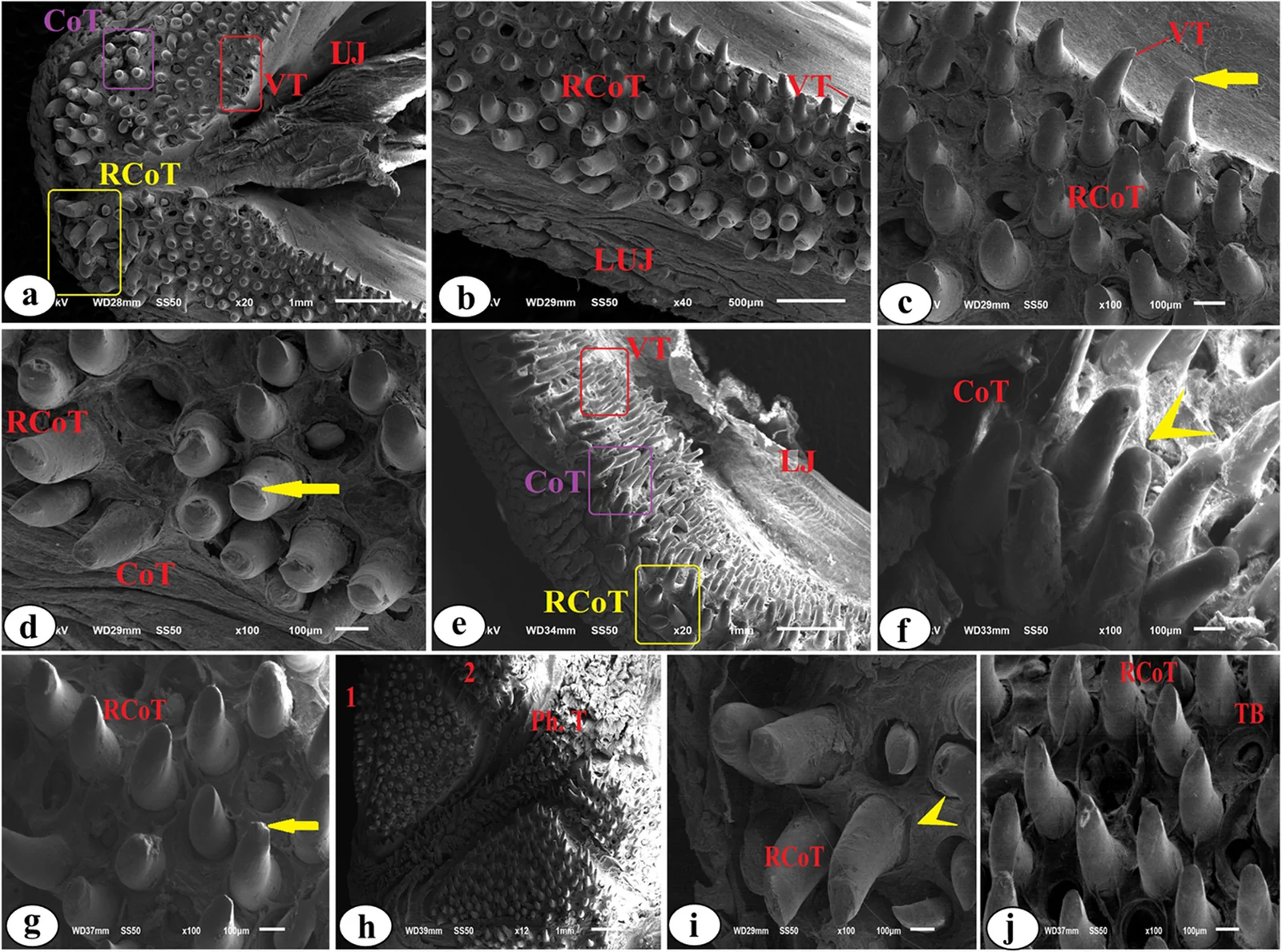
Scanning electron micrographs of S. spiniferum teeth showing: (**a-g**): upper jaw (UJ) and lower jaw (LJ) with multiple rows of teeth; the anterior middle were more pominent conical or “caniniform” (hooked or fang like) (CoT). Also, sharp conical, pointed recurved inward (RCoT), and numerous, fine and needle-like villiform teeth (VT), arranged in dense patches along the edges of both the upper and lower jaws. The teeth edges were sharp and microserrations or ridges found on cutting surfaces (**red head arrow**). Microgrooves, microridges and irregularities found at the surface (**yellow head arrows**). (**h-j**) Sharp and conical pharyngeal teeth similar to jaw teeth and arranged in two pads; (upper, 1) and (lower, 2), it were (CoT) and may be recurved (RCoT). Note: the presence of microgrooves at the bases of teeth (**yellow head arrow**) and taste buds (TB)

**Fig. 16:**
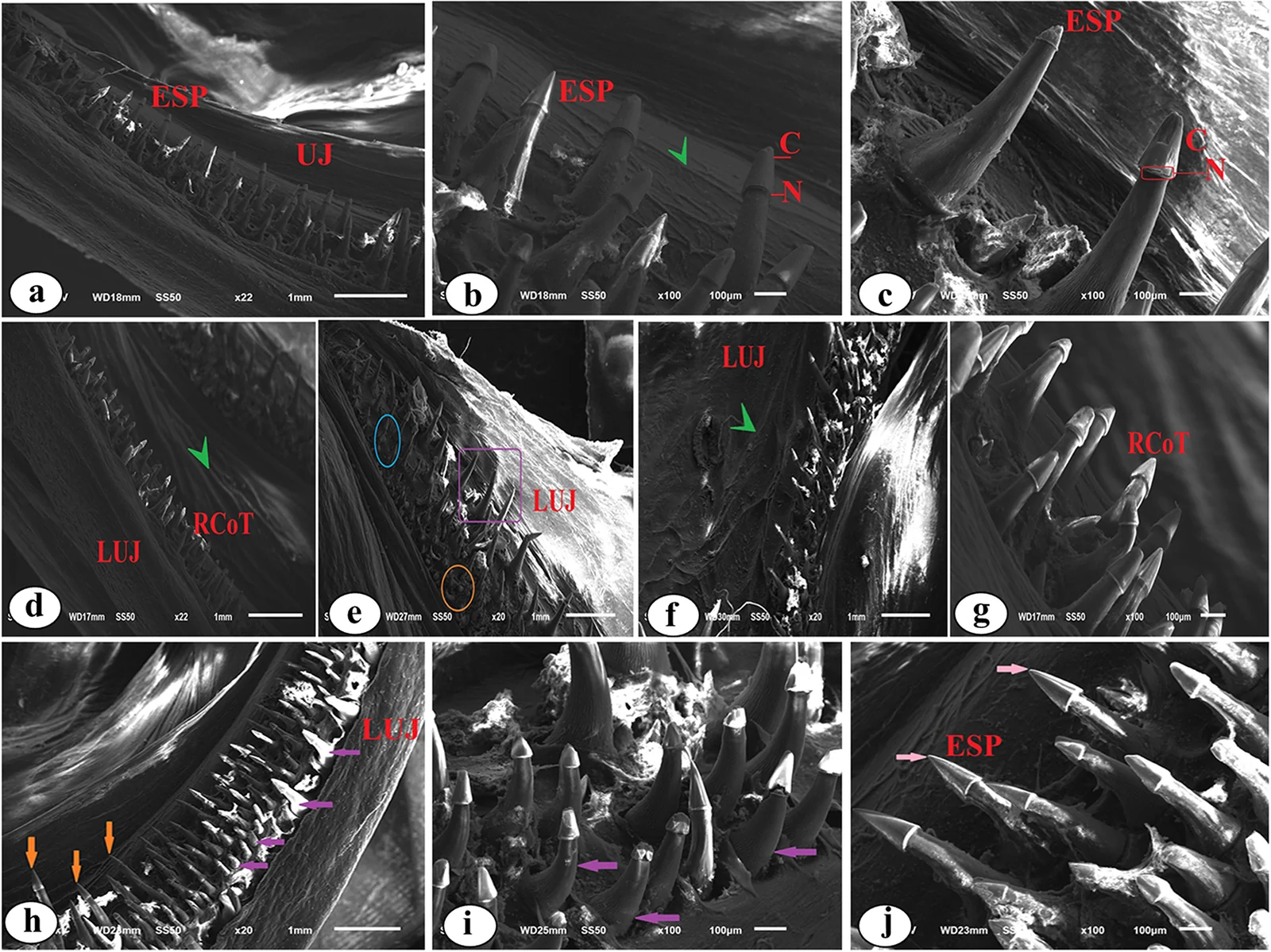
(**a-j**): scanning electron micrographs of *C. miniata* upper jaw (UJ) appeared with microriges (green head arrows) showing: multiple rows of conical teeth; (**a-c**) The anterior middle were more elongated sharp pointed (ESP) with hook like process has crown (**C**) and prominent neck region (**N**). (**d-j**) lateral upper jaw (LUJ) has conical teeth which may appeared straight (blue circle), recurved outer short (orange circle, RCoT, violet arrows), or recurved inner elongated (violet rectangle, orange arrows). Note: all these teeth have hook like processes (pink arrows)

Rabbit-fishes revealed two types of teeth; tri-cusped (Fig. 14a–c) and bi-cusped (Fig. 14d, e), which were organized in a single row on the jaws. Tricuspid teeth were spatula- or chisel-shaped with slight curvature, closely spaced, and equipped with sharp, serrated edges and micro-ridges for scraping algae. The pharyngeal teeth, located on the roof of the buccal cavity and were divided into two halves, each with three pads (upper, middle, and lower) containing needle-like, flattened, or hook-shaped teeth with basal protuberances for wear resistance (Fig. 14f–i). *S. obtusata* had a large, protractile mouth with a lower jaw that protruded noticeably and a powerful array of long, sharp-edged, pointed teeth resembling piranha dentition (Fig. 7d). Upper posterior teeth were sharp and recurved for grasping and piercing prey, while the two bilateral pairs of upper anterior teeth lined the tip of the premaxilla. These teeth were large, strong canines that protruded from the symphysis connecting the two bilateral elements and fit neatly in a recess between the anterior most canines of the upper jaws to impale and hold slippery prey, frequently extending outside the closed mouth (Fig. 7e, f). A single median tall, powerful, and sharp canine anterior tooth (Fig. 7g) was carried at the tip of the lower jaw’s midline. It resembled upper anterior teeth but was positioned on the front of the lower jaw to interlock with upper anterior teeth to prevent prey from escaping, potentially aiding in the manipulation or holding of prey. As prey moves farther into the mouth, the lower posterior prominent teeth in the back of the lower jaw help with secondary grip (Fig. 7h). The maxilla was impaled by short, straight, serrated teeth with a truncate edge (Fig. 7i). These teeth were all caniniform, or fang-like, sharp conical blades that were designed to pierce soft-bodied prey in order to prevent escape by interlocking mechanisms. Pharyngeal teeth appeared as two halves and featured small, sharp, conical teeth that resembled spines, were adapted for a predatory lifestyle and helped with gripping and manipulating prey rather than grinding (Fig. 15a). In the dorsal pharyngeal region, teeth that were grouped together and had smooth enamel surfaces exhibited extremely sharp cutting edges with microserrations (Fig. 15b–f). It had villiform teeth coated in layers of enameloid over dentin as a potentially crystalline structure (Fig. 16c–e), and the lateral pharyngeal surfaces showed minute ridges or microgrooves (Fig. 16a, b). Supporting pharyngeal tissue was made up of fibrous connective tissue, which had a dense mesh of interlacing collagen fibers (Fig. 16f). This tissue gave pharyngeal teeth mechanical support and anchorage, enabling strong fixation, resistance to mechanical stress during prey handling, and flexibility without tooth loss.

**Fig. 17:**
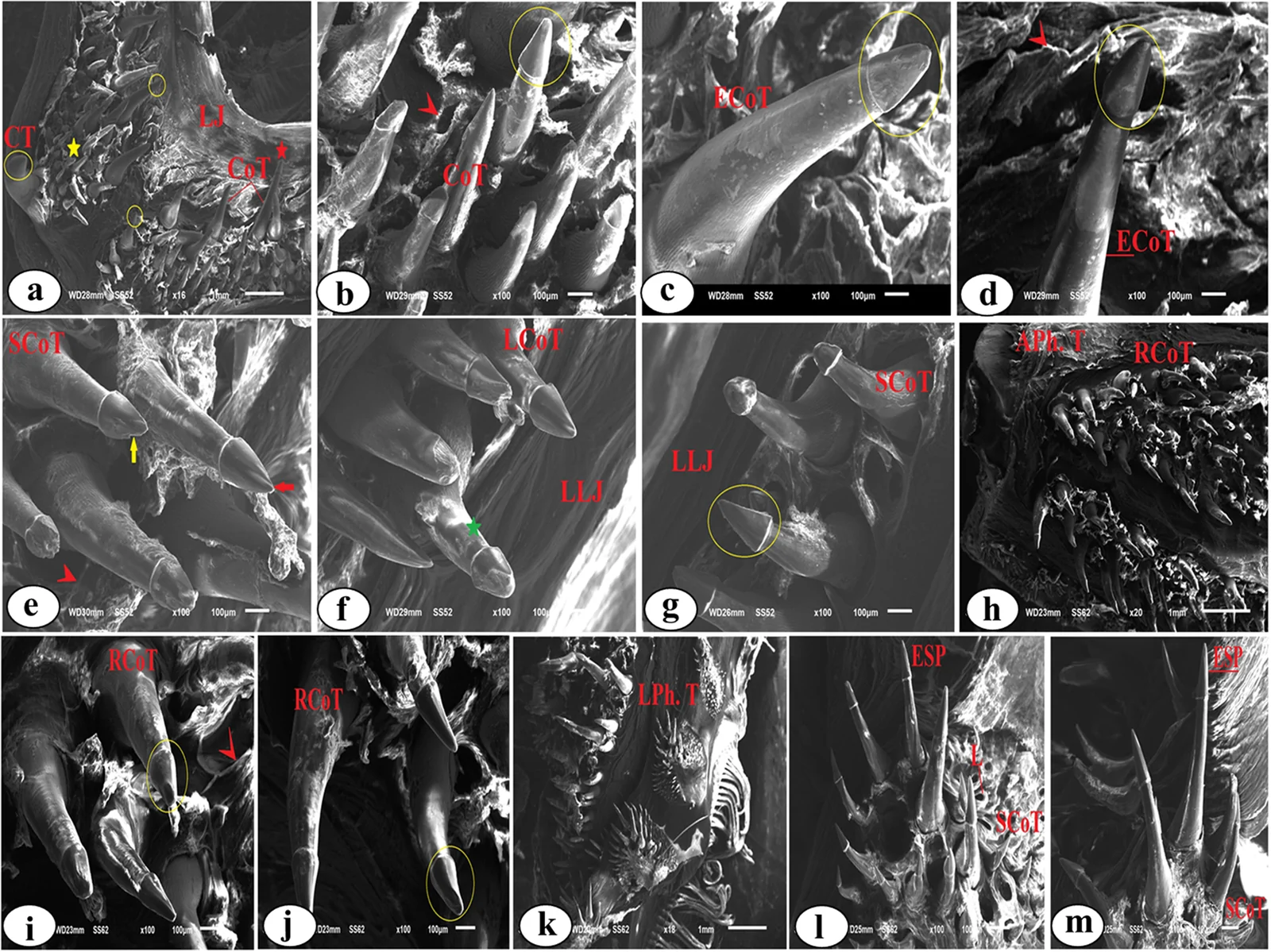
Scanning electron micrographs of *C. miniata* showing: (**a-e**) middle lower jaw (LJ) showing pominent anterior caniniform fang‑like teeth (CT), conical (CoT) may appeared anterior short (**yellow star, SCoT**) or posterior elongated (**red star, ECoT**), all these teeth appeared with hook (**yellow circle**) that may appeared with smooth surface (**red arrow**) or with little serrations (**yellow arrows**). Note: microgrooves and irregularities found at the surface (**red arrow head**). (**f, g**) lateral lower jaw (LLJ) has conical hooked teeth either long (LCoT) or small (SCoT). (**h-m**) Scanning electron micrographs of *C. miniata* pharyngeal teeth showing its arrangement either in anterior rows (APh.T) appeared recurved (RCoT) or laterally in groups or patches appeared needle shape as elongated sharp pointed (ESP) and small (SCoT). Note: the presence of ligament (**L**) To anchor pharyngeal teeth together

Long-jawed *S. spiniferum* had four types of teeth; prominent anterior canine teeth that were used to crush shellfish (Fig. 17a), molariform teeth that were present posteriorly for crushing (Fig. 17b), and small, sharp, conical teeth that were arranged in bands of multiple rows along the strong upper and lower jaws as well as on the roof of the mouth (Palatines) (Fig. 17c). Additionally, the vomer had a V-shaped area of vomerine teeth and palatine that were tightly spaced in dense bands or patches that resembled brush bristles (Fig. 17a–g). Instead of piercing or cutting, the mouth and tooth structure were suited for biting, grabbing, probably crushing, and suction feeding. They were also designed to hold and grip small, slick prey like fish or invertebrates, which is consistent with the function of villiform teeth. The teeth’s edges had serrations, grooves, and microridges (Fig. 17c, d, f). The pharyngeal teeth were divided into two halves, each of which had two pads with small, sharp, conical teeth for grasping and processing prey such as worms and crustaceans (Fig. 17h–j). These teeth help with swallowing and manipulating prey rather than grinding it.

The *C. miniata* had a large mouth with several rows of teeth (Fig. 16b, c); the anterior were more pominent conical or caniniform (hooked or fang‑like). Additionally, the margins of both the upper and lower jaws had dense patches of many, thin, needle-like villiform teeth that were sharp, conical, pointed, and recurved inward (Fig. 16d–f). Predatory fish that had evolved to hold slippery prey, such as smaller fish or invertebrates, instead of tearing or crushing, frequently have this kind of dentition. Upper jaw appeared with microriges showed multiple rows of conical teeth; the anterior middle was more elongated sharp pointed with hook like process has crown and prominent neck region (Fig. 16a–c). Lateral upper jaw has conical teeth which may appear straight, recurved outer short, or recurved inner elongated with hook like processes (Fig. 16d–j). Middle lower jaw showed pominent anterior caniniform fang‑like teeth, conical may appear anterior short or posterior elongated, all these teeth appeared with hook that may appeared with smooth surface or with little serrations. Microgrooves and irregularities found at the surface (Fig. 17a–e). Lateral lower jaw had conical hooked teeth either long or small (Fig. 17f, g). The edges of all teeth were sharp, and as patterns of wear and polishing, there were microserrations or ridges on cutting surfaces, microgrooves, and imperfections as adaptations for improving grip on slip prey (Fig. 17d, e). Similar to jaw teeth, pharyngeal teeth were sharp and conical. They were employed to grasp and retain prey, such as fish and crabs, and to help manipulate them rather than grind them. It seemed recurved when grouped in anterior rows (Fig. 17h–j) or needle-shaped when arranged laterally in groups or patches (Fig. 17k–m). In order to prevent the prey from escaping back through the anterior jaws, the ligament at the bases of the pharyngeal teeth aided in their anchoring together and in depressing the teeth as the prey approached the throat.

**Fig. 18:**
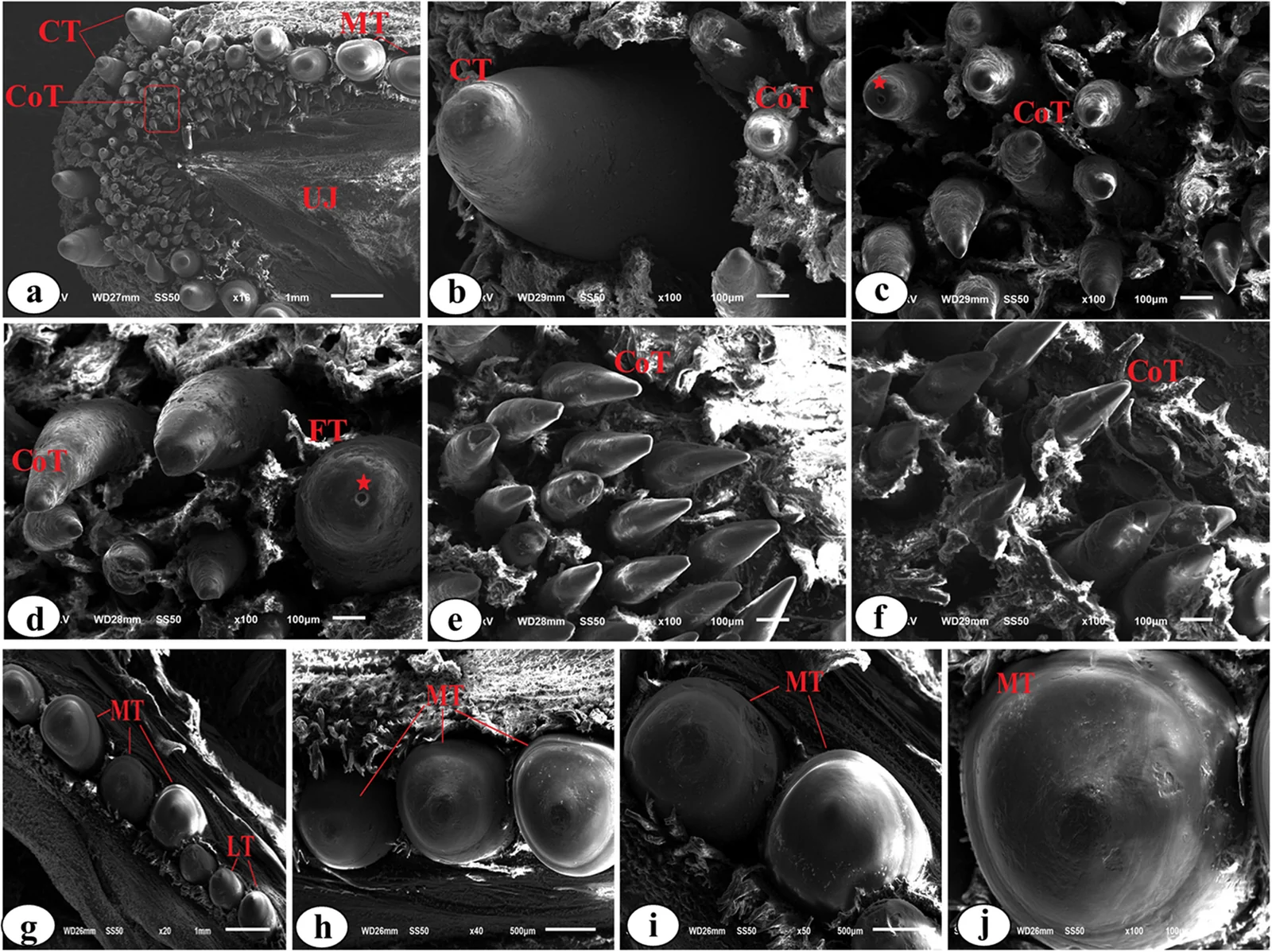
(**a-j**) Scanning electron micrographs of L. lentjan upper jaw (UJ) showing: anterior prominent conical canine teeth (CT), conical teeth (CoT), broad blunt rounded molariform (MT), and vomerine lateral teeth in other parts at the back (LT). Microgrooves or striations found on the enameloid surface, especially on edges of teeth. Note: flattened teeth (FT) found among conical and beared taste buds (**red star**)

**Fig. 19:**
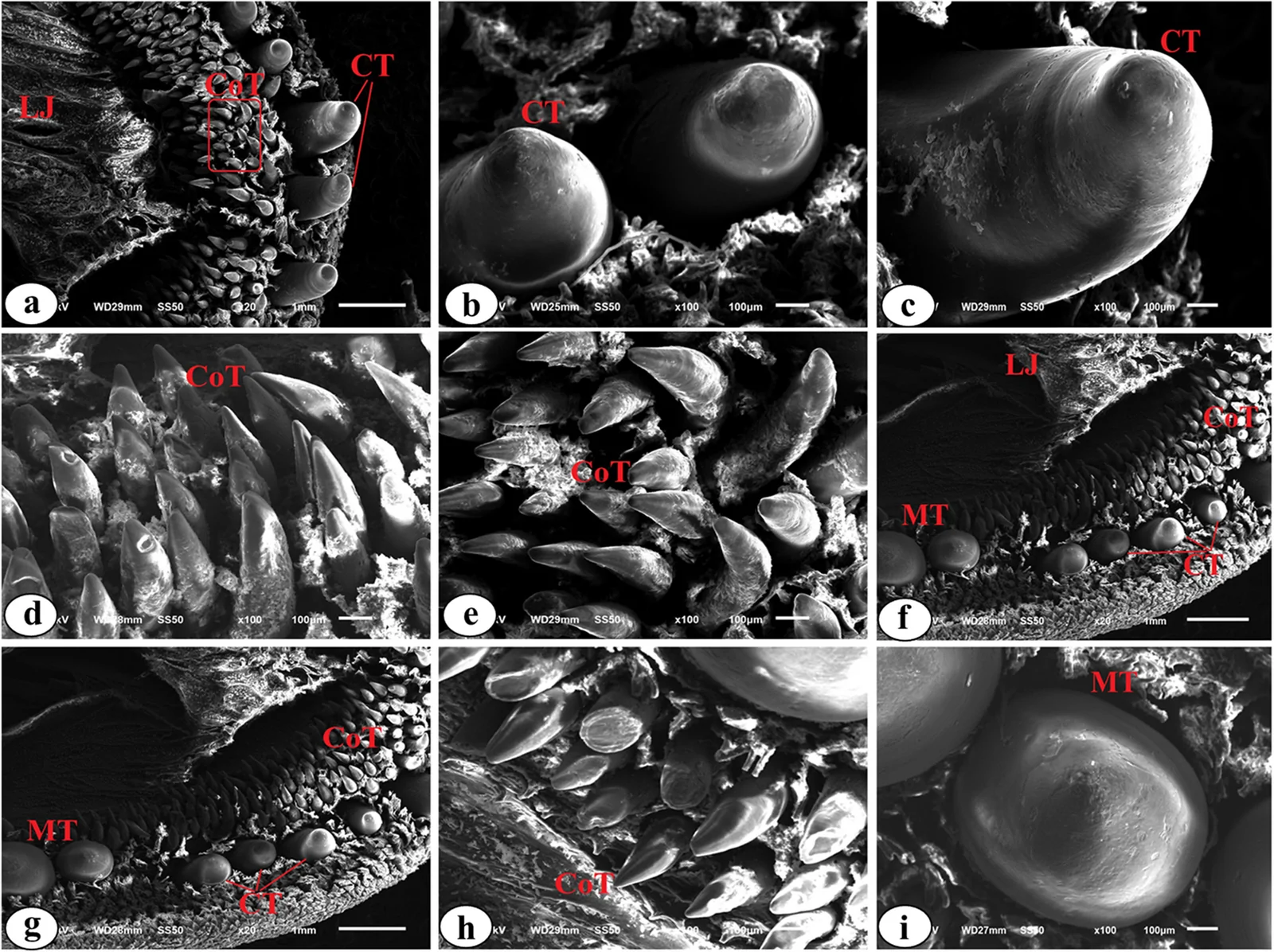
(**a-i**) Scanning electron micrographs of L. lentjan lower jaw (LJ) showing: anterior single row of canine teeth (CT), posterior conical teeth (CoT) arranged in rows, and posterior molariform round teeth (MT)

**Fig. 20:**
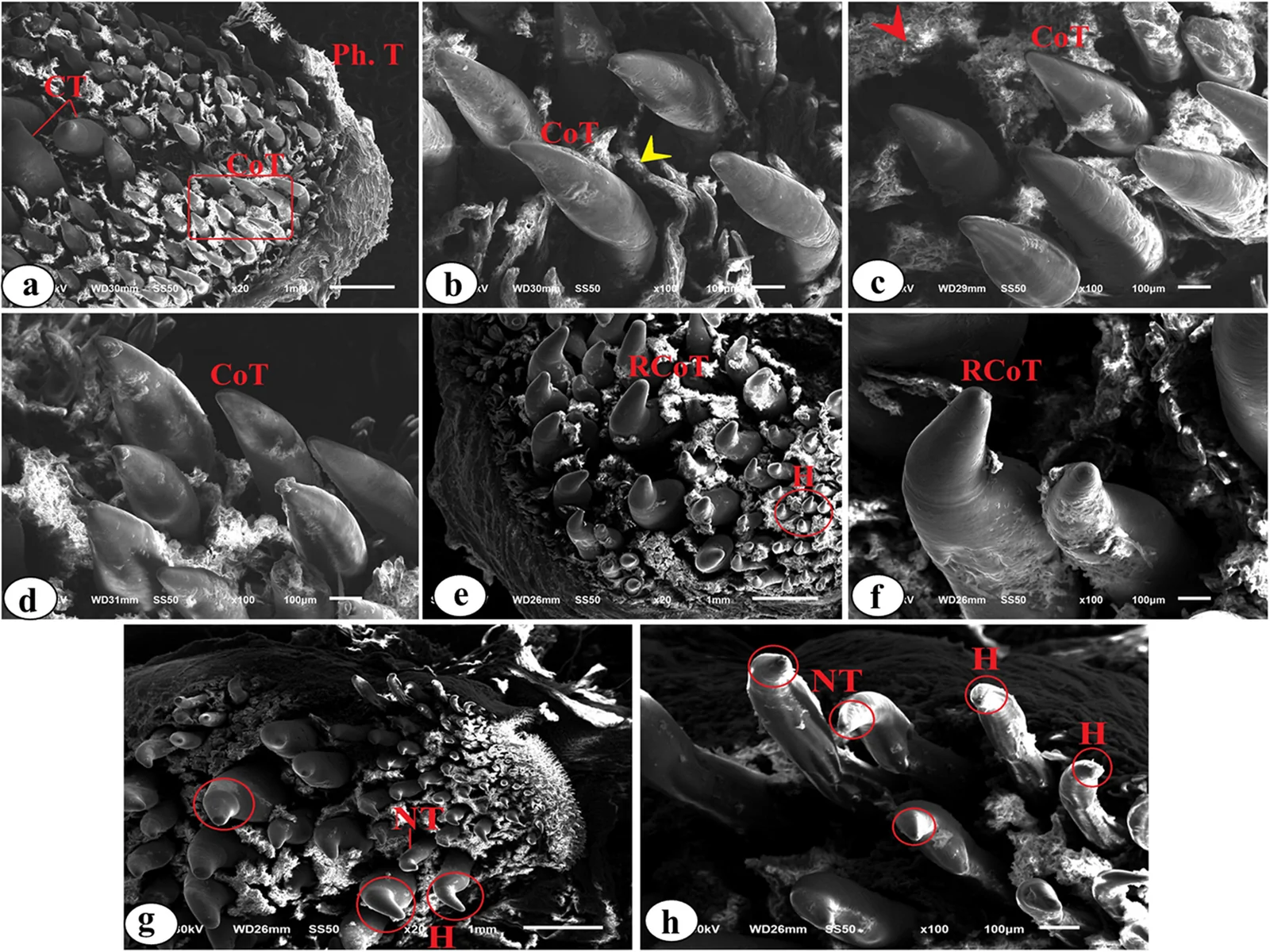
(**a-h**) Scanning electron micrographs of L. lentjan pharyngeal teeth (Ph. T) showing: (**a-d**) anterior pharyngeal teeth has conical and pointed teeth (CoT) in front and canine teeth appeared posteriorly (CT). (**e, f**) lateral pharyngeal teeth has recurved conical (RCoT) appeared with hooks (red circle, H). (**g, h**) posterior pharyngeal teeth appeared in batches of elongated needle has hooks (**H**)

The upper jaw of *L. lentjan* displayed anterior prominent canine teeth (Fig. 18a, b), conical teeth, vomerine lateral teeth in other parts at the back, and flattened teeth found among conical and beared taste buds (Fig. 18c–f). Molariform teeth that were broad, blunt, and rounded were located posteriorly (Fig. 18g–j). The lower jaw displayed anterior single row of canine teeth (Fig. 19a–c), posterior conical teeth organized in rows (Fig. 19d–f), and posterior molariform round teeth (Fig. 19g–i). Pharyngeal teeth had anterior conical and pointed teeth and canine teeth posteriorly (Fig. 20a–d). Recurved conical lateral pharyngeal teeth with hooks were visible (Fig. 20e, f). The posterior pharyngeal teeth had hooks and appeared in patches of long needles (Fig. 20g, h). The enameloid surface has microgrooves or striations, particularly on the edges of teeth. Parts of teeth have smooth enamel to reduce wear. Canine teeth have sharp points or cutting edges, while crushing teeth have more rounded or flattened surfaces. Additionally, vomerine teeth had patches (Figs. 18, 19 and 20).

### GeMoMa-based gene prediction and filtering

**Fig. 21:**
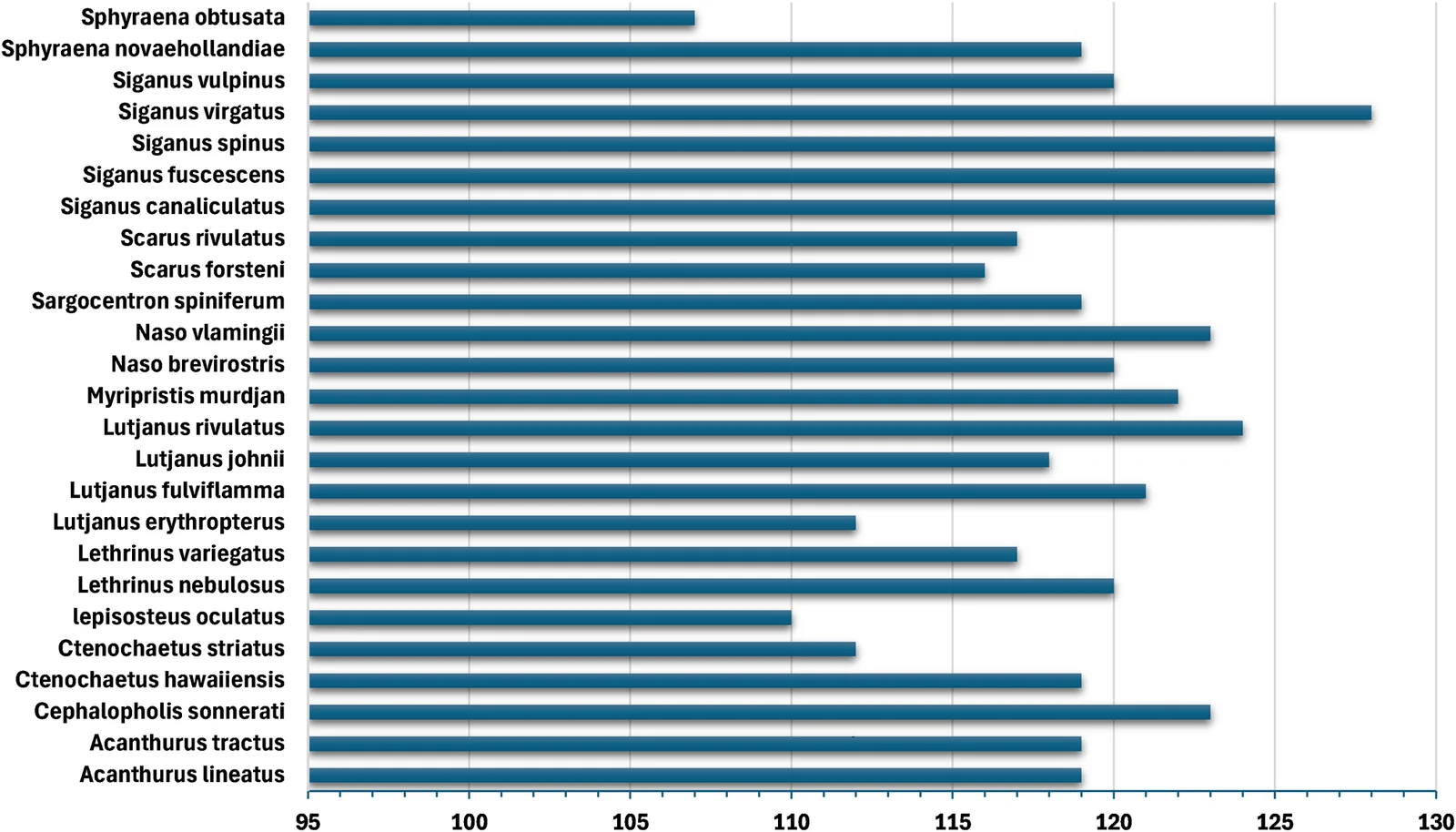
Number of recovered tooth-associated genes per species following prediction and filtering

**Fig. 22:**
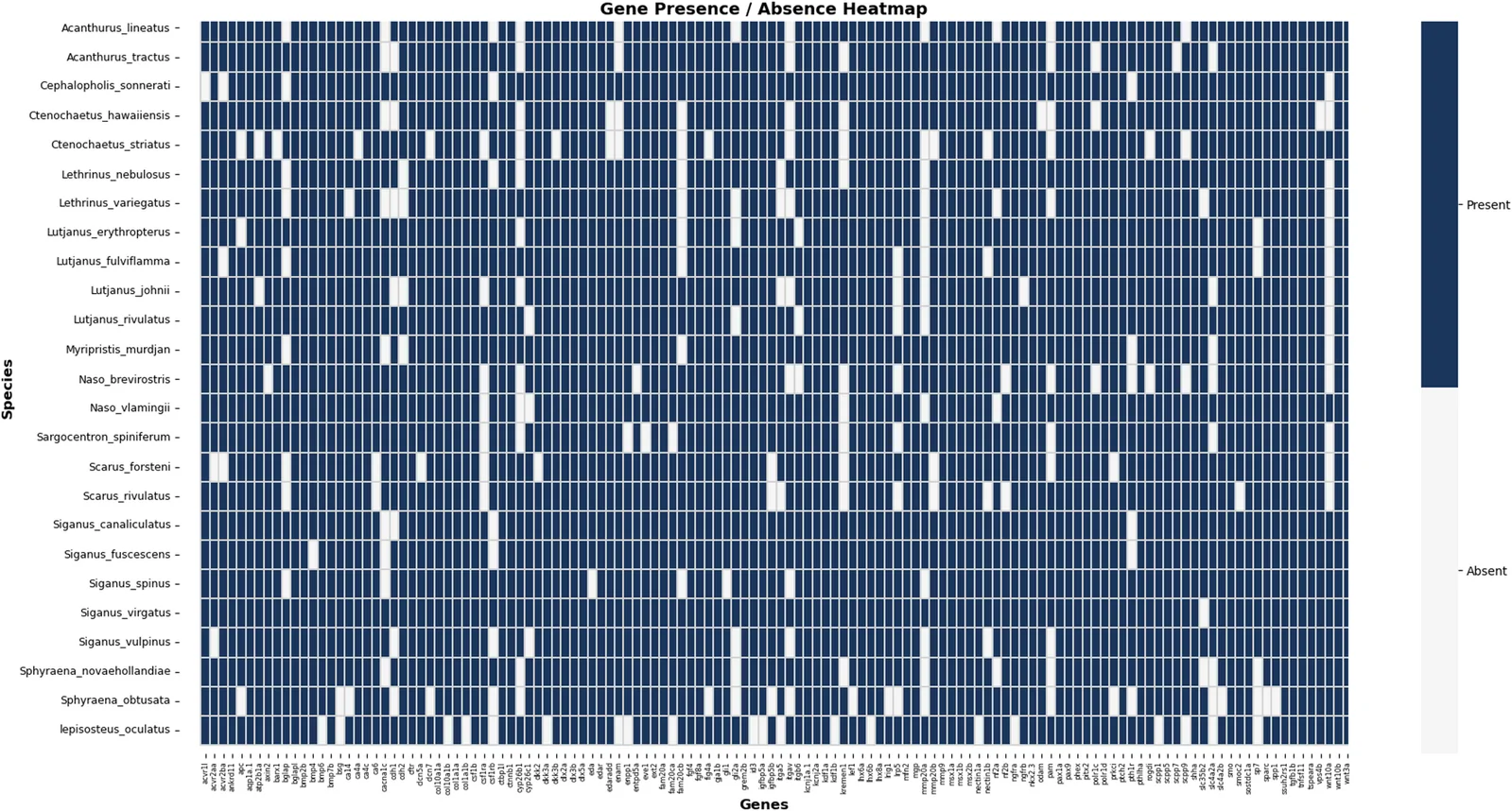
Heatmap showing the recovery of tooth-associated genes across the analyzed species

Gene recovery was consistently high across the 25 analyzed genomes, including the outgroup, following merging and filtering steps (Fig. 21). The number of recovered loci per species ranged from 107 to 128 out of 130 candidate loci, with an average of 119.2 genes per genome. Most species retained more than 115 loci, indicating strong conservation of tooth-associated loci across teleost lineages. The highest recovery was observed in *Siganus virgatus* (128 genes), whereas *Sphyraena obtusata* showed the lowest recovery (107 genes). The outgroup *Lepisosteus oculatus* showed moderate recovery with 110 genes. More than 90% of the candidate loci were recovered in the majority of species, supporting the robustness of the homology-based annotation pipeline. After homology-based prediction and GAF filtering, many tooth-associated genes were highly conserved among the analyzed teleost genomes. Of the 130 candidate loci, 52 genes were detected in all species (25/25), including ankrd11, aqp1a.1, bmp2b, bmp7b, dlx2a, dlx3b, fgf4, fgf8a, and shha (Fig. 22). A second group of 59 genes was nearly ubiquitous, occurring in 21–24 species, while a smaller subset of 20 genes, present in 12–20 species, suggesting partial or lineage-specific retention that may reflect gene loss, sequence divergence, or incomplete genome assemblies.

### Phylogenetic relationships and evolutionary patterns

**Fig. 23:**
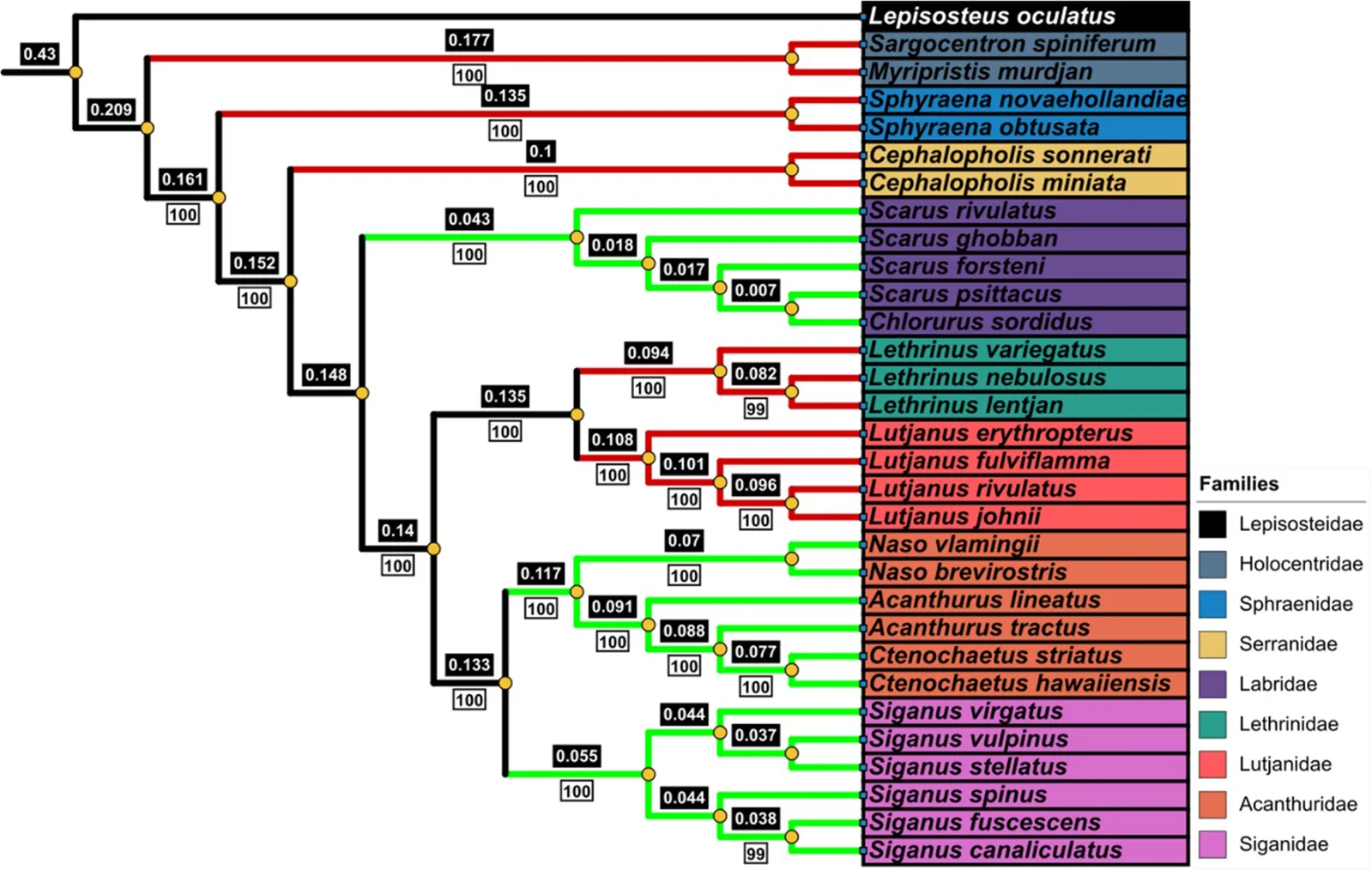
Maximum-likelihood phylogenetic tree inferred from concatenated, partitioned protein alignments of 130 tooth-associated genes across 31 species. Branch support was assessed with 1,000 ultrafast bootstrap replicates. Bootstrap support values are shown in white boxes at the nodes, whereas branch lengths are displayed in black boxes. Branch colors indicate dietary category: carnivorous lineages are shown in red and herbivorous lineages in green

The maximum-likelihood phylogeny recovered a well-resolved topology with strong statistical support across major nodes (Fig. 23). The out-group, *Lepisosteus oculatus*, occupied a basal position, providing a clear root for the tree and establishing the direction of evolutionary divergence among the remaining taxa. Ingroup species, primarily representing Perciform lineages, clustered largely according to family-level taxonomy. Each family formed a well-supported monophyletic clade with bootstrap values generally reaching 100%, including Holocentridae, Sphyraenidae, Serranidae, Labridae, Lethrinidae, Lutjanidae, Siganidae, and Acanthuridae. This pattern indicates that tooth-associated genes retain a strong phylogenetic signal consistent with shared ancestry and vertical inheritance.

When dietary categories were mapped onto the phylogeny, carnivorous (red branches) and herbivorous (green branches) species formed coherent clusters within their respective families. Herbivorous lineages, such as Siganidae, Acanthuridae, and herbivorous members of Labridae, grouped into well-supported clades, while carnivorous families including Serranidae, Lutjanidae, Sphyraenidae, and Lethrinidae also formed distinct clusters. These patterns suggest that the evolution of tooth-development genes broadly parallels taxonomic divergence. Within families, species diversification likely reflects divergence from a common ancestor while maintaining similar feeding strategies. Stochastic character mapping estimated an average of 2.64 state transitions across the phylogeny, including a mean of 1.44 transitions from carnivory to herbivory and 1.20 transitions from herbivory to carnivory across 1,000 simulations. These results indicate repeated evolutionary shifts in trophic strategy among the sampled lineages.

Variation in branch lengths was observed across the phylogeny, reflecting differences in sequence divergence among lineages. Together with the stochastic character mapping analysis, the phylogeny indicates that carnivorous and herbivorous feeding strategies are distributed across multiple teleost lineages while preserving the expected family-level phylogenetic relationships.

### Gene-wide episodic selection (BUSTED)

Among the 130 analyzed tooth-associated genes, 74 showed significant evidence of episodic positive selection under the BUSTED model. After applying false discovery rate (FDR) correction, 70 genes remained statistically significant, indicating widespread episodic diversification across teleost lineages. Several genes with well-established roles in odontogenesis were retained after correction, including gli2a, smoc2, cdh2, eda, and acvr1l. Additional regulators associated with odontogenesis, such as csf1ra, csf1rb, and tspeara, also exhibited significant signals of selection. Notably, multiple components of the Wnt signaling pathway involved in tooth morphogenesis, apc, dkk2, dkk3b, wnt10b, and wnt6b, displayed strong evidence of episodic selection, highlighting the evolutionary flexibility of key regulatory pathways underlying dental development.

### Branch-specific episodic selection (aBSREL)

**Fig. 24:**
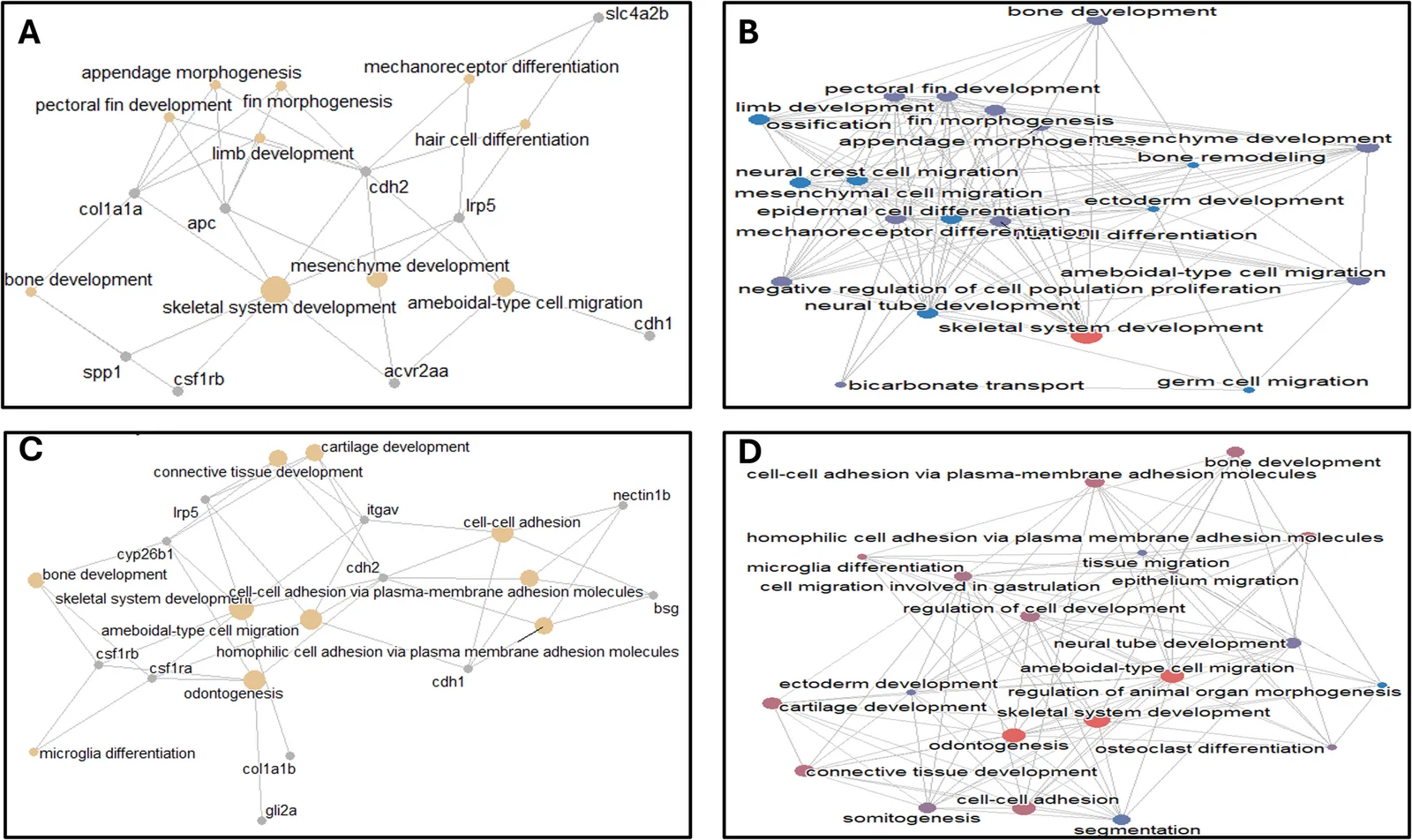
Functional enrichment of genes under episodic selection in herbivorous and carnivorous lineages. (**A**–**B**) herbivorous species: enrichment map (emapplot) and gene–concept network (cnetplot). Node size reflects the number of gene–function interactions, with larger nodes indicating more connected GO terms. (**C**–**D**) carnivorous species: enrichment map and gene–concept network, where node size similarly represents the extent of gene–function interactions

In herbivorous lineages, significant episodic selection was detected in several genes, including nectin1b, cdh1, csf1rb, lrp5, clcn5a, col1a1a, spp1, slc4a2b, cdh2, acvr2aa, nf2a, apc, ca4a, pam, cftr, and pth1r. Functional enrichment analysis indicated that these genes were primarily associated with processes related to skeletal and craniofacial development, including skeletal system development, bone development, bone mineralization, ossification, biomineral tissue development, and odontogenesis. Additional enriched categories involved mesenchymal development, neural crest cell migration, epithelial differentiation, and cell–cell adhesion, all of which are key processes underlying tooth formation and mineralized tissue remodeling. Notably, several enriched pathways were also linked to Wnt signaling regulation, appendage and fin morphogenesis, and tissue regeneration, suggesting that genes under selection in herbivorous species may contribute to structural remodeling of feeding-related skeletal and dental tissues. The relationships between enriched biological processes and the associated genes are summarized in Fig. 24A–B.

In carnivorous lineages, episodic selection was detected in a partially overlapping but distinct set of genes, including cdh2, lrp5, cyp26b1, cdh1, col1a1b, itgav, csf1rb, pam, bglapl, ngfrb, bsg, ngfra, nectin1b, csf1ra, gli2a, and mmp20a. Enrichment analyses revealed strong associations with biological processes related to odontogenesis, cartilage and skeletal development, connective tissue formation, and bone remodeling, as well as pathways involved in cell migration, neural crest development, and epithelial morphogenesis. Several enriched functions were also linked to axon guidance, neural patterning, and sensory system development, reflecting the broader developmental roles of these genes in craniofacial and oral structures. Gene–function relationships for carnivorous lineages are illustrated in Fig. 24C–D.

Despite dietary differences, a subset of genes including cdh2, cdh1, lrp5, csf1rb, pam, and nectin1b showed signals of selection in both herbivorous and carnivorous lineages. These shared genes are primarily involved in cell adhesion, skeletal morphogenesis, and tooth development, suggesting that they represent core components of conserved odontogenic pathways that remain evolutionarily dynamic across trophic strategies. Together, these results indicate that while many tooth-associated genes evolve under lineage-specific selective pressures linked to feeding ecology, a core set of developmental regulators continues to experience adaptive diversification across teleost diets.

### Mixed effects model of evolution (MEME) analysis

**Fig. 25:**
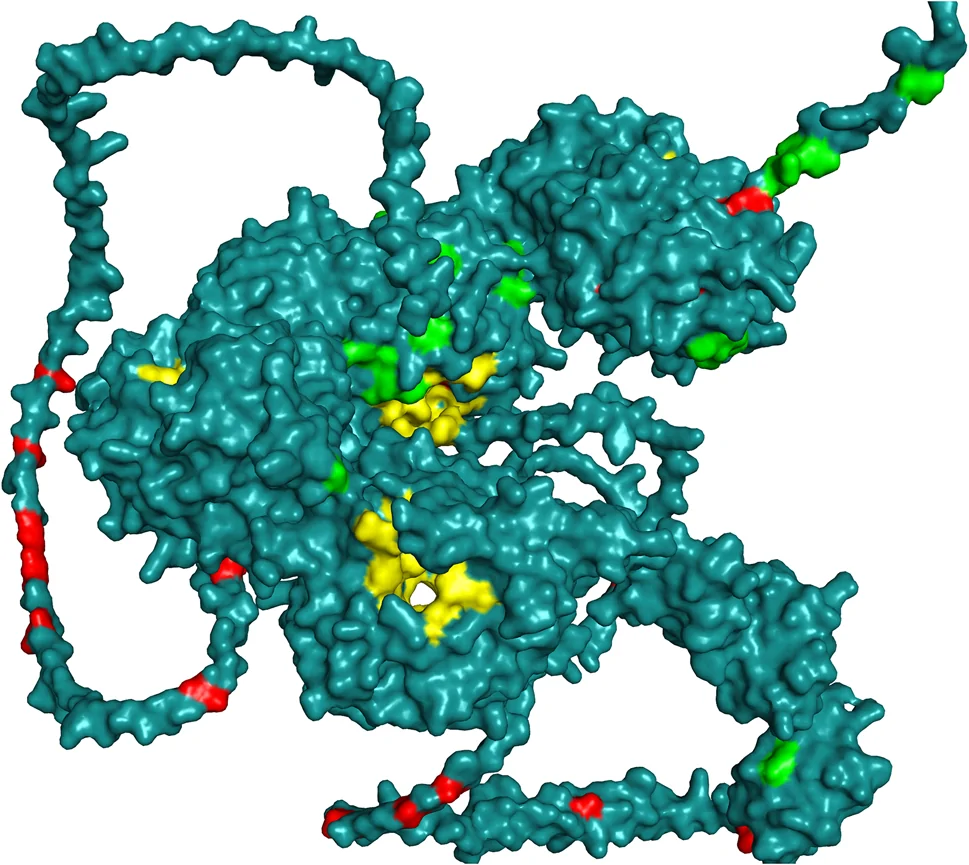
Structural mapping of codon sites under episodic positive selection in lrp5. The predicted three-dimensional protein structure shows codons under episodic selection in herbivorous lineages highlighted in green, codons under selection in carnivorous lineages in red, and predicted active-site residues in yellow. Several selected sites (258, 259, 396, 409, and 438) overlap with predicted active-site residues, suggesting potential functional relevance of adaptive amino acid substitutions

**Fig. 26:**
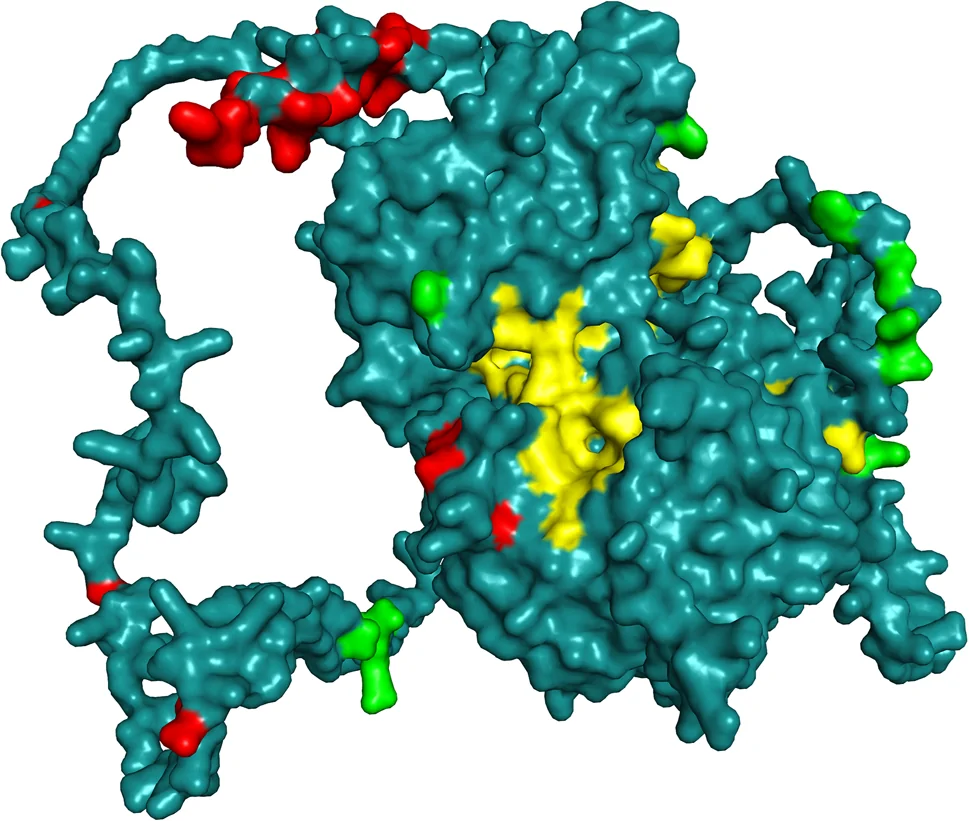
Structural mapping of codon sites under episodic positive selection in pam. The predicted three-dimensional protein structure highlights codons under episodic selection in herbivorous lineages (green), codons under selection in carnivorous lineages (red), and predicted active-site residues (yellow). Overlapping positions between selected codons (373, 375, and 411) and active-site residues indicate possible functional effects of adaptive changes in the protein

The analyses revealed multiple codon sites under episodic selection across the six genes in both dietary groups (Table 3). To explore their potential functional relevance, these codons were compared with predicted ligand-binding residues derived from P2rank. Notably, in two genes lrp5 and pam several codons under episodic selection in herbivorous lineages overlapped with predicted active-site residues. Specifically, selected codons were detected at positions 258, 259, 396, 409, and 438 in lrp5, and at positions 373, 375, and 411 in pam. Mapping these residues onto predicted three-dimensional protein structures revealed that some positively selected sites are located within or near functional regions of the proteins. In the structural visualizations, codons under selection in herbivorous lineages are shown in green, those under selection in carnivorous lineages in red, and predicted active-site residues in yellow (Figs. 25, 26). This spatial overlap suggests that adaptive amino acid substitutions in these proteins may influence functional properties related to their biological roles.

**Table 3:** Codon sites under episodic positive selection detected by the MEME model for the six genes shared between herbivorous and carnivorous lineages

Gene	Foreground	Episodic Selection	herb vs carn	Overlap with predicted active sites
cdh1	herbivore	25, 61, 62, 72, 78, 79, 85, 100, 129, 152, 153, 156, 170, 185, 189, 192, 193, 212, 216, 218, 223, 225, 242, 259, 261, 297, 303, 304, 313, 358, 416, 448, 479, 618, 626, 653, 808, 828	313	-
	carnivore	8, 9, 12, 13, 15, 17, 29, 38, 39, 49, 51, 52, 63, 88, 112, 244, 248, 268, 313, 314, 374, 399, 409, 424, 452, 499, 581, 604, 630, 665, 678, 679, 681, 683, 856		
cdh2	herbivore	4, 11, 20, 21, 22, 23, 24, 25, 30, 32, 33, 43, 44, 46, 48, 126, 187, 503, 509, 524, 528	4, 32	-
	carnivore	3, 4, 15, 32, 145, 641		
csf1rb	herbivore	11, 37, 181, 290, 307, 332, 354, 366, 372, 374, 378, 408, 426, 505, 508, 521, 535, 580, 587, 612, 772, 780, 782, 785, 789, 804, 933, 986, 989, 991, 992, 994, 995, 997, 998, 1000, 1004, 1007, 1009, 1020, 1034	11, 374, 426	-
	carnivore	1, 6, 9, 11, 42, 51, 126, 135, 156, 162, 212, 248, 269, 340, 371, 374, 414, 424, 426, 428, 450, 486, 553, 556, 569, 597, 693, 951, 1027, 1031, 1032, 1037, 1039		
lrp5	herbivore	4, 19, 20, 23, 24, 206, 239, 254, 257, 258, 259, 261, 349, 367, 368, 385, 396, 398, 409, 416, 417, 418, 419, 435, 438, 673, 952, 1072, 1359	23, 24	258, 259, 396, 409, 438
	carnivore	16, 17, 23, 24, 67, 333, 576, 986, 1325, 1398, 1403, 1408, 1410, 1412, 1428, 1451, 1456, 1458, 1493, 1567, 1570, 1575, 1583, 1597, 1600, 1601, 1602, 1603, 1607, 1611		-
nectin1b	herbivore	11, 203, 336, 342, 455, 460, 472		-
	carnivore	2, 3, 4, 11, 12, 17, 259		
pam	herbivore	28, 39, 69, 84, 366, 373, 375, 376, 388, 411, 418, 470, 471, 472, 482, 807, 808, 835, 838, 841, 854, 858, 859, 862, 967	28, 471	373, 375, 411
	carnivore	2, 8, 9, 10, 12, 15, 16, 18, 25, 27, 28, 29, 30, 32, 61, 98, 274, 337, 397, 432, 435, 436, 471, 522, 799, 817, 848, 878, 893, 896		-

Analyses were conducted separately using herbivorous and carnivorous branches as foreground lineages. Codon positions with *p* < 0.1 are reported together with dietary comparison and their overlap with predicted active-site residues.

**Fig. 27:**
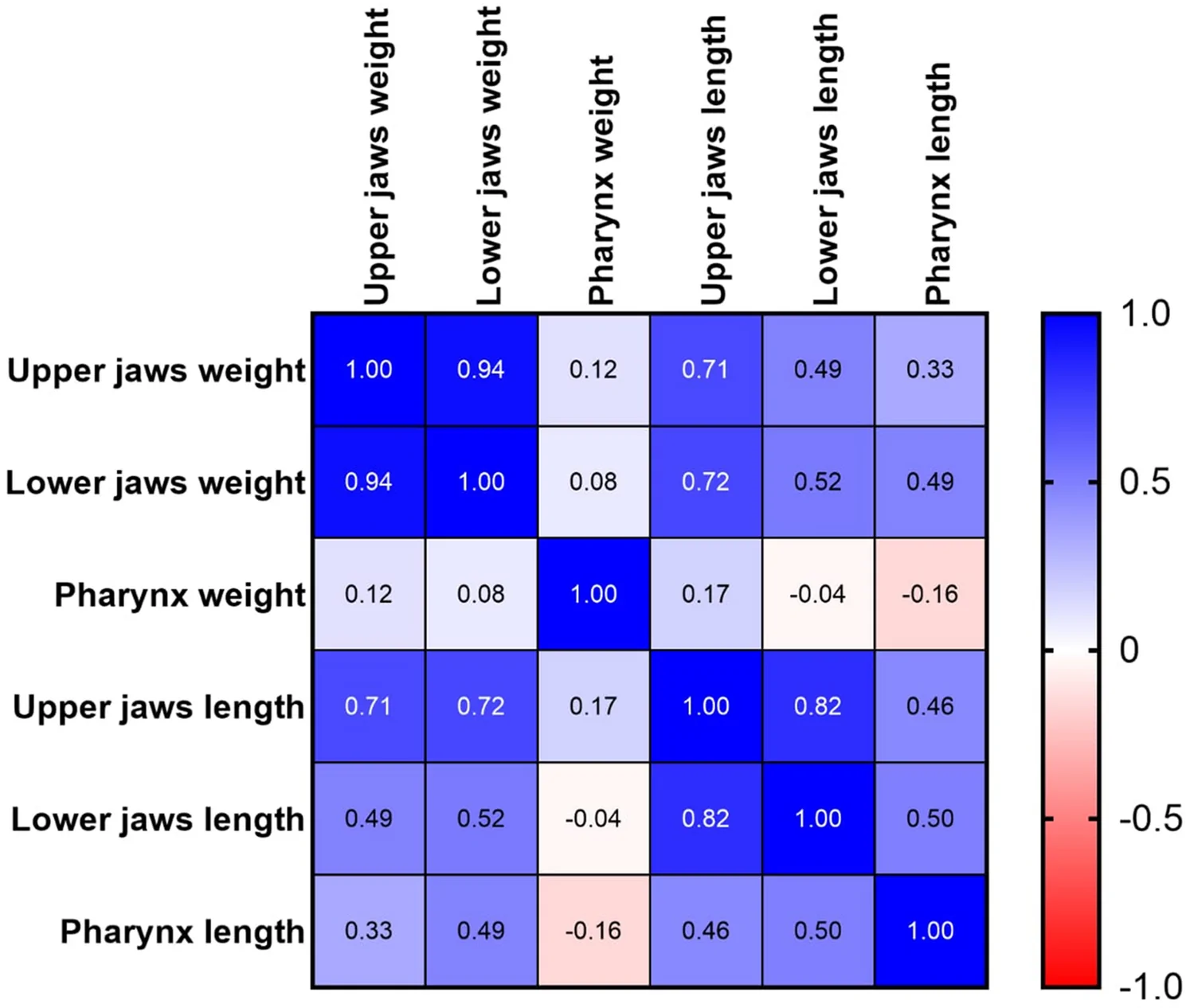
Pearson correlation of size-standardized gross morphological measurements in the studied fish species

**Fig. 28:**
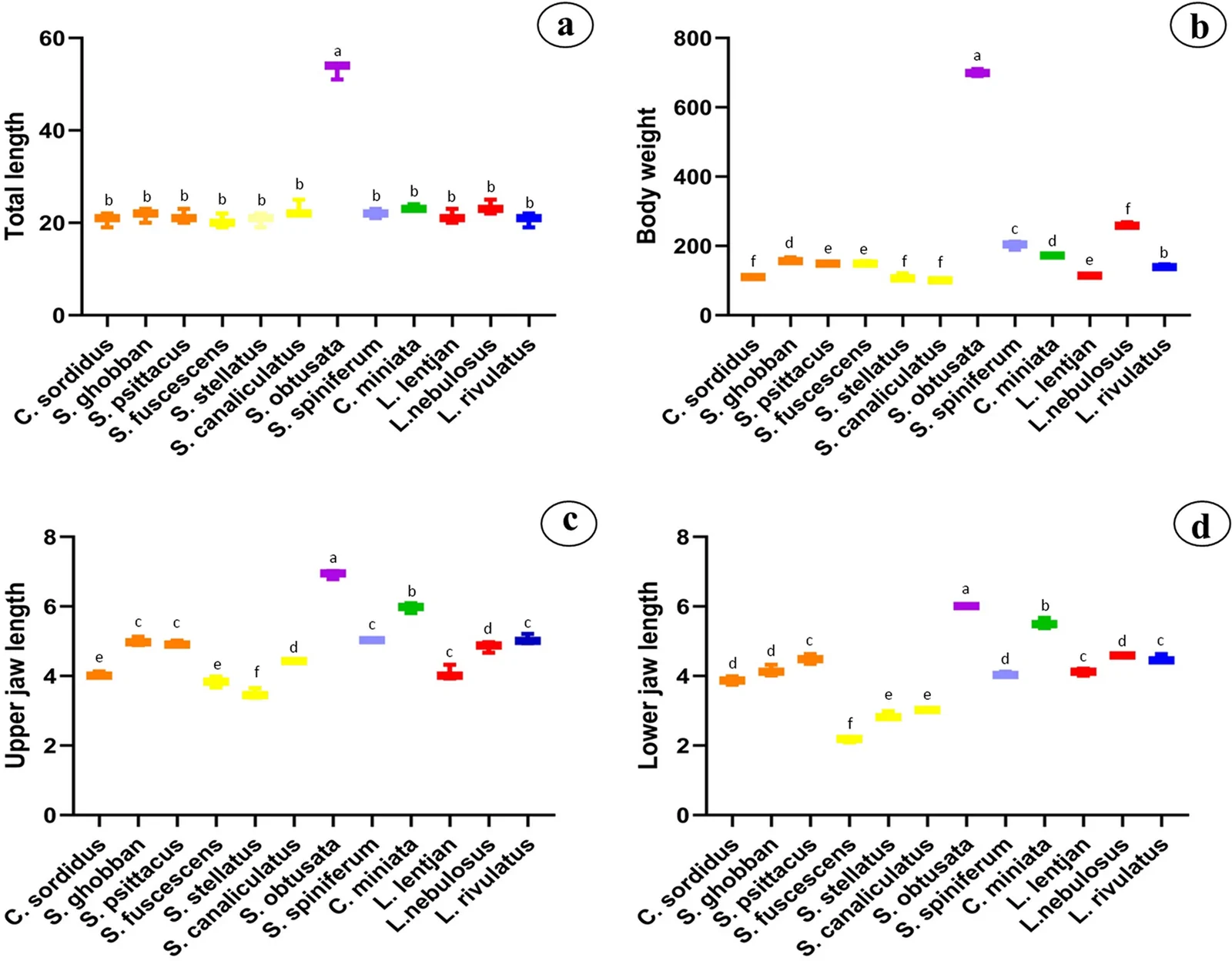
Morphometric parameters of fish species in Marsa Alam, Red Sea. (**a**) Total length (cm), (**b**) Body weight (g), (c) Upper jaw length (cm), and (**d**) Lower jaw length (cm). Data are presented as mean ± SD. a, b, c, d, e and f refer to the significance of each letter compared to the following letters. Means that do not share a letter are significantly different (Tukey’s test, p < 0.05)

### Relaxed selection test (RELAX)

Several genes showed selection intensification in herbivorous lineages and relaxation in carnivorous lineages (Table 4), including csf1rb, cdh2, spp1, slc4a2b, mmp20a, edar, phex, pax9, tspeara, and ankrd11. Many of these genes are involved in biological processes related to odontogenesis, ossification, skeletal system development, and skeletal morphogenesis. In contrast, another subset of genes exhibited the opposite pattern, with relaxation in herbivores and intensification in carnivores, including cdh1, gli2a, dkk3b, bglapl, nf2b, bsg, scpp9, lhx8a, and fgf4, which are associated with processes such as odontogenesis, fibroblast growth factor signaling, and axonogenesis. These results suggest that herbivorous and carnivorous lineages experience different selective pressures on distinct developmental pathways related to tooth and craniofacial biology.

**Table 4:** Results of the RELAX analysis for tooth-associated genes

Gene	K	RELAX_Herb	K	RELAX_Carn
csf1rb	4.785	Intensification	0.206	Relaxation
cdh2	1.236	Intensification	0.716	Relaxation
spp1	1.533	Intensification	0.648	Relaxation
slc4a2b	4.085	Intensification	0.373	Relaxation
mmp20a	2.579	Intensification	0.238	Relaxation
edar	3.681	Intensification	0.251	Relaxation
phex	5.005	Intensification	0.168	Relaxation
polr1c	4.107	Intensification	0.275	Relaxation
edaradd	3.619	Intensification	0.268	Relaxation
ca6	3.655	Intensification	0.303	Relaxation
ca4c	1.463	Intensification	0.663	Relaxation
enpp1	1.868	Intensification	0.536	Relaxation
ptch2	1.643	Intensification	0.748	Relaxation
acvr1l	6.371	Intensification	0.040	Relaxation
pax9	5.787	Intensification	0.148	Relaxation
tspeara	2.541	Intensification	0.264	Relaxation
ankrd11	1.173	Intensification	0.820	Relaxation
nkx2.3	5.984	Intensification	0.204	Relaxation
tnfsf11	3.104	Intensification	0.315	Relaxation
sostdc1a	10.152	Intensification	0.091	Relaxation
smo	4.984	Intensification	0.673	Relaxation
scpp5	3.833	Intensification	0.232	Relaxation
igfbp5b	3.611	Intensification	0.245	Relaxation
mfn2	6.782	Intensification		
ctbp1l	5.599	Intensification		
scpp1	1.608	Intensification		
polr1d	12.122	Intensification		
cdh1	0.428	Relaxation	5.788	Intensification
gli2a	0.453	Relaxation	1.953	Intensification
dkk3b	0.445	Relaxation	2.249	Intensification
bglapl	0.235	Relaxation	4.499	Intensification
nf2b	0.203	Relaxation	4.857	Intensification
bsg	0.629	Relaxation	1.589	Intensification
vps4b	0.627	Relaxation	5.761	Intensification
scpp9	0.102	Relaxation	3.621	Intensification
lhx8a	0.000	Relaxation	13.958	Intensification
fgf4	0.572	Relaxation	1.725	Intensification
ext2	0.000	Relaxation	5.823	Intensification
lrig1	0.504	Relaxation	3.467	Intensification
cyp26c1	0.000	Relaxation	4.147	Intensification
ngfrb	0.167	Relaxation	0.322	Relaxation
cyp26b1	0.000	Relaxation	0.265	Relaxation
wnt10a	0.000	Relaxation	0.000	Relaxation
kdf1a	0.000	Relaxation	0.000	Relaxation
clcn5a	0.594	Relaxation	0.000	Relaxation
enam	0.000	Relaxation	5.106	Intensification
wnt5a	0.000	Relaxation		
igfbp5a	0.422	Relaxation		

The table reports the gene name, the selection intensity parameter (K) estimated by RELAX, and the inferred selection regime (intensification or relaxation) when herbivorous or carnivorous lineages were treated as foreground branches. Values of K > 1 indicate selection intensification, whereas K < 1 indicate selection relaxation.

## Discussion

Fishes have evolved to have a variety of teeth to adapt their life depending on their ecology, diets, and phylogenetic relationships, this variation can be seen in the teeth of the upper and lower jaws as well as the pharynx. A striking example of ecological zonation, where habitat partitioning acted as a key mechanism for preserving ecosystem stability, was provided by the complex spatial distribution of the investigated species within the Marsa Alam marine province. Through niche partitioning, this distribution successfully reduced direct competition; similar observations were reported by [12, 36]. According to [37, 38], the high concentration of Siganidae and Scaridae in shallow-water zones (1–30 m) suggests a specialized reliance on high-light environments. These species serve as essential functional herbivores that maintain reef integrity by grazing on the epilithic algal matrix, thereby preventing algal dominance over coral recruits. This process is identified as the main driver behind the assembly of coral reef fish communities. Families like Lethrinidae (emperors) and Lutjanidae (snappers), which feed on a wide variety of prey, from mollusks to small fish, preserve the equilibrium of invertebrate populations in the transitional zones (5–60 m). On the other hand, a trophic bridge between the reef and the open marine environment (200 m) is facilitated by the presence of Sphyraenidae as a high-level predator operating across deeper and pelagic reaches, which has been reported as crucial for energy transfer across diverse marine habitats by [14, 39].

An evolutionary pressure for biomechanical efficiency and specialized ecological roles is reflected in the significant variation in size-independent jaw and body metrics found in our standardized data (*p* < 0.001). For example, in line with findings by [13], the elongated, shape standardized jaws of *Sphyraena obtusata* represent a distinct piscivorous morphotype that is biomechanically optimized for ram-feeding, showcasing the highest jaw to body length ratio among the studied group. This species is strongly segregated along the positive axis of PC1 (accounting for 57.32% of total variance), driven predominantly by jaw lengths and weights. According to [9, 10], this specialized morphological trait serves as an ecological adaptation for high speed pursuit and enhanced capacity to capture large, mobile prey in open water [15], independent of absolute body size.

These sympatric fish species effectively avoid trophic niche overlap by divergent diversification of their oral jaw structures, pharyngeal mill configurations, and specialized dentition. The size-standardized Principal Component Analysis (PCA) confirms that these species are morphologically segregated into distinct functional guilds, such as *Siganus* species being aligned with pharyngeal weight on PC2 (19.07%) which is a structural indicator of a stable and mature ecosystem. The Marsa Alam marine environment functions as a robust and highly productive biological system precisely due to this ecological specialization, which guarantees that all available benthic and pelagic energy sources from microscopic endolithic algae to macro-invertebrates and fish are efficiently exploited across differentiated trophic levels.

A complex network of size-independent morphometric interdependencies, reflecting a precise functional and biological integration among the parameters under study, was further elucidated by the Pearson correlation analysis (Fig. 4). In stark contrast to unstandardized configurations where body mass confounds phenotypic covariance, the adjusted matrix demonstrated a highly integrated oral jaw module. This modularity is characterized by strong, size-independent coupling between upper and lower jaw weights (*r* = 0.94) and lengths (*r* = 0.82). From an ecomorphological standpoint, these robust correlations suggest a localized structural integration strategy that aims to synchronize structural components within the primary capture apparatus to ensure optimal mechanical leverage and biting efficiency during foraging, a pattern that aligns conceptually with baseline modular evolutionary models [13].

On the other hand, the adjusted dataset revealed a significant functional decoupling particularly regarding the pharyngeal weight from the anterior oral jaw apparatus. This observation is consistent with the functional variations seen in advanced reference studies on teleost cranial evolution. The Pharynx weight demonstrated remarkably weak to negligible correlations with both oral jaw weights (*r* = 0.08 to 0.12). This evolutionary dissociation implies that the pharyngeal mill follows a distinct developmental and functional trajectory independent of the primary oral premaxillary and mandibular structures. Whereas pharynx length retains moderate coupling with jaw dimensions (*r* = 0.46 to 0.50).

This macro-evolutionary autonomy increases the organism’s overall ecological resilience by acting as a defensive mechanism to preserve vital physiological functions such as pharyngeal respiration and deglutition as a biological survival priority under changing environmental pressures or fluctuations in localized food availability [40]. Consequently, the trophic apparatus is partitioned into distinct, localized functional units; an oral module for prey acquisition and an independent pharyngeal module optimized for mechanical processing.

The herbivorous species of family Scaridae (Parrotfish) had incisors that were modified to the shape of a saw-edge or fused into the beak suited for scraping at the coral and shredding or cutting algae, as well as large flat-topped grinding teeth at the back of its throat for crushing the coral and algal mixture. This result was consistent with the previous data of [12, 41], but it differed with the viper fish results reported by [42]. The herbivorous species of family Siganidae had smaller and lighter jaws with tricuspid or bicuspid teeth reflect an adaptation for rapid, repetitive nipping, this allows them to clean the reef surfaces effectively, preventing algal overgrowth that could otherwise smother living corals, this result was parallel with [11, 43]. Both dorsal and pharyngeal teeth appeared needle shape and gradually curve in the caudal direction, they also formed hooks at their tips to aid in food ingestion, this result was parallel with the previous study of [44, 45].

Conversely, the carnivorous species belonging to the families Sphyraenidae, Holocentridae, Serranidae, Lethrinidae, and Lutjanidae have a combination of dentition types, either long canines combined with smaller conical teeth or anterior canines followed by or mixed with tiny needlelike teeth. The majority of carnivores have canine teeth, which are long, cone-shaped teeth that can be straight or curved. They are used to pierce, puncture, and hold prey as well as feed on small fish and crustaceans, these results were parallel with previous data of [46, 47]. According to research reported by [48], the family Sphyraenidae used a flat-bladed, pointed, triangular dentition to cut off prey. Similar findings were noted in the Holocentridae family, which has long, sharp teeth that are used to grasp fish [49]. The Serranidae family has many small, needle-like villiform teeth that repeat on the palatine or vomerine bones. The teeth that pointed backward were elongated, very long, and fine, and they were used for stabbing and direction. They may have ligamentous connections at their base that allowed them to be depressed as the prey moved toward the throat but prevented escape back through the anterior jaws [50, 51], confirmed the previous data. The families Lethrinidae and Lutjanidae had recurved, conical, caniniform teeth with sharp points for gripping as well as flattened or rounded broad molariform teeth found posteriorly used for crushing and grinding foods like mollusks, these findings were consistent with [8, 52, 53].

The pharyngeal teeth, which are located in the throat and are made up of dorsal and ventral pads, are arranged in several rows of depressed teeth that produce a functionally flat surface that is more suited for grabbing onto and crushing their hard food. After a bite, these teeth spring back up. The pharyngeal teeth of Serranidae and Lethrinidae have ligaments at their bases that allowed them to hinge backwards, overlapping the replacement tooth that sits immediately behind the jaws, these results were in accordance with data reported by [54, 55]. 

Our analyses reveal pervasive adaptive evolution in teleost tooth-associated genes. BUSTED tests identified episodic positive selection in ~ 70 of 130 loci after FDR correction, indicating that adaptive diversification is superimposed on a deeply conserved odontogenic gene network previously thought to be largely stable [56]. Many positively selected genes belong to core developmental pathways, including Wnt, BMP, FGF, and EDA. Experimental evidence demonstrates the central roles of these pathways in tooth formation and regeneration: Wnt10a promotes tooth replacement, whereas bmp6 and the Wnt antagonist dkk2 inhibit it, and both are essential for normal tooth morphogenesis [57]. Similarly, Eda signaling is critical for proper odontogenesis and can induce ectopic teeth when overexpressed [58], explaining why these pathways are recurrent targets of positive selection.

In addition to signaling regulators, strong selection on extracellular matrix and biomineralization genes (e.g., col1a1, col10a1, spp1, mmp20, enam) suggests that remodeling of tooth tissues represents a major axis of dental diversification. This mirrors patterns in mammals, where adaptive evolution in enamel- and dentin-related genes enhances resistance to abrasive diets [59, 60]. Selection on FGF pathway components further supports experimental findings that modulation of FGF/BMP signaling can generate supernumerary or morphologically complex teeth, illustrating that subtle regulatory changes can produce variation in tooth number and shape [61].

Branch-specific a BSREL analyses revealed trophic signatures: herbivorous lineages exhibited intensified selection on bio-mineralization and craniofacial genes (lrp5, spp1, col1a1), whereas carnivorous lineages showed selection on alternate developmental regulators (gli2a, mmp20a), consistent with ecological divergence driving dental evolution [59, 60]. MEME analyses indicated that adaptive substitutions often occur at functionally relevant codons, while RELAX results confirmed stronger selection intensity in herbivores on matrix and ossification genes and in carnivores on signaling components [62, 63].

These findings demonstrate that teleost tooth genes are both conserved and evolutionarily labile, with repeated adaptive targeting of signaling pathways (Wnt, BMP, FGF, EDA) and matrix components across vertebrates. This highlights the odontogenic gene network as a flexible evolutionary toolkit underlying dental diversity and reinforces the principle that modifications in key developmental and structural genes drive convergent trophic adaptations in vertebrate dentitions.

## Conclusion

This study demonstrates that Red Sea fishes from Marsa Alam maintain ecosystem balance through diversified feeding strategies, with herbivorous and carnivorous species occupying distinct ecological niches. Size-standardized morphological analyses revealed clear evolutionary segregation and modular integration between jaw mechanics and pharyngeal processing apparatuses, fully supported by robust PCA configurations and high-resolution SEM observations. Herbivorous taxa exhibited specialized scraping dentition adapted for algal feeding in shallow zones, whereas carnivorous species displayed grasping and piercing teeth suited for predation across broader depth ranges. The integration of comparative genomics with morphological data provided deeper insight into the evolutionary mechanisms underlying this diversity. Phylogenetic analyses supported strong taxonomic structuring alongside independent origins of herbivory, while selection analyses revealed widespread adaptive evolution in tooth-associated genes. Contrasting selection pressures between trophic groups suggest that dietary specialization has shaped distinct molecular pathways, particularly those related to odontogenesis and skeletal development in herbivores and growth factor and neural processes in carnivores. Site level evidence of adaptive changes in functionally relevant regions further supports the role of molecular evolution in shaping dental traits. These findings highlight a strong link between ecological function, tooth morphology, and gene evolution, emphasizing how trophic adaptation drives both phenotypic and genomic diversification in teleost fishes.

## Electronic supplementary material

Below is the link to the electronic supplementary material.

Supplementary Material 1

## Data Availability

Due to our institute’s rules, the datasets generated and analyzed during the current study are not publicly available, but they are available from the corresponding Author upon reasonable request.
